# Genetic–epigenetic interplay in the determination of plant 3D genome organization

**DOI:** 10.1093/nar/gkae690

**Published:** 2024-08-16

**Authors:** Xiaoning He, Chloé Dias Lopes, Leonardo I Pereyra-Bistrain, Ying Huang, Jing An, Rim Brik Chaouche, Hugo Zalzalé, Qingyi Wang, Xing Ma, Javier Antunez-Sanchez, Catherine Bergounioux, Sophie Piquerez, Sotirios Fragkostefanakis, Yijing Zhang, Shaojian Zheng, Martin Crespi, Magdy M Mahfouz, Olivier Mathieu, Federico Ariel, Jose Gutierrez-Marcos, Xingwang Li, Nicolas Bouché, Cécile Raynaud, David Latrasse, Moussa Benhamed

**Affiliations:** Université Paris-Saclay, CNRS, INRAE, Univ Evry, Institute of Plant Sciences Paris-Saclay (IPS2), Orsay 91405, France; Université Paris-Saclay, CNRS, INRAE, Univ Evry, Institute of Plant Sciences Paris-Saclay (IPS2), Orsay 91405, France; Université Paris-Saclay, CNRS, INRAE, Univ Evry, Institute of Plant Sciences Paris-Saclay (IPS2), Orsay 91405, France; Université de Paris Cité, Institute of Plant Sciences Paris-Saclay (IPS2), 91190 Gif-sur-Yvette, France; Université Paris-Saclay, CNRS, INRAE, Univ Evry, Institute of Plant Sciences Paris-Saclay (IPS2), Orsay 91405, France; Université Paris-Saclay, CNRS, INRAE, Univ Evry, Institute of Plant Sciences Paris-Saclay (IPS2), Orsay 91405, France; Université Paris-Saclay, CNRS, INRAE, Univ Evry, Institute of Plant Sciences Paris-Saclay (IPS2), Orsay 91405, France; Université Paris-Saclay, CNRS, INRAE, Univ Evry, Institute of Plant Sciences Paris-Saclay (IPS2), Orsay 91405, France; Université de Paris Cité, Institute of Plant Sciences Paris-Saclay (IPS2), 91190 Gif-sur-Yvette, France; Université Paris-Saclay, CNRS, INRAE, Univ Evry, Institute of Plant Sciences Paris-Saclay (IPS2), Orsay 91405, France; Université Paris-Saclay, CNRS, INRAE, Univ Evry, Institute of Plant Sciences Paris-Saclay (IPS2), Orsay 91405, France; School of Life Science, University of Warwick, Coventry CV4 7AL, UK; Université Paris-Saclay, CNRS, INRAE, Univ Evry, Institute of Plant Sciences Paris-Saclay (IPS2), Orsay 91405, France; Université Paris-Saclay, CNRS, INRAE, Univ Evry, Institute of Plant Sciences Paris-Saclay (IPS2), Orsay 91405, France; Department of Biosciences, Molecular Cell Biology of Plants, Goethe University Frankfurt am Main, Max-von-Laue Str. 9, 60438 Frankfurt am Main, Germany; State Key Laboratory of Genetic Engineering, Collaborative Innovation Center of Genetics and Development, Department of Biochemistry, Institute of Plant Biology, School of Life Sciences, Fudan University, Shanghai 200438, China; State Key Laboratory of Plant Physiology and Biochemistry, College of Life Sciences, Zheijang University, Hangzhou 310058, China; Université Paris-Saclay, CNRS, INRAE, Univ Evry, Institute of Plant Sciences Paris-Saclay (IPS2), Orsay 91405, France; Division of Biological and Environmental Sciences and Engineering, King Abdullah University of Science and Technology, Thuwal 23955-6900, Kingdom of Saudi Arabia; Institute of Genetics Reproduction and Development (iGReD), Université Clermont Auvergne, CNRS, Inserm, Clermont-Ferrand, F-63000, France; Universidad de Buenos Aires (UBA), Facultad de Ciencias Exactas y Naturales, and Instituto de Fisiología, Biología Molecular y Neurociencias (IFIBYNE), CONICET-UBA, Buenos Aires, Argentina; School of Life Science, University of Warwick, Coventry CV4 7AL, UK; National Key Laboratory of Crop Genetic Improvment, Hubei Hongshan Laboratory, Huazhong Agricultural University, Wuhan 430070 Hubei, China; Université Paris-Saclay, INRAE, AgroParisTech, Institute Jean-Pierre Bourgin for Plant Sciences (IJPB), 78000 Versailles, France; Université Paris-Saclay, CNRS, INRAE, Univ Evry, Institute of Plant Sciences Paris-Saclay (IPS2), Orsay 91405, France; Université Paris-Saclay, CNRS, INRAE, Univ Evry, Institute of Plant Sciences Paris-Saclay (IPS2), Orsay 91405, France; Université Paris-Saclay, CNRS, INRAE, Univ Evry, Institute of Plant Sciences Paris-Saclay (IPS2), Orsay 91405, France; Université de Paris Cité, Institute of Plant Sciences Paris-Saclay (IPS2), 91190 Gif-sur-Yvette, France; Institut Universitaire de France (IUF), Orsay, France

## Abstract

The 3D chromatin organization plays a major role in the control of gene expression. However, our comprehension of the governing principles behind nuclear organization remains incomplete. Particularly, the spatial segregation of loci with similar repressive transcriptional states in plants poses a significant yet poorly understood puzzle. In this study, employing a combination of genetics and advanced 3D genomics approaches, we demonstrated that a redistribution of facultative heterochromatin marks in regions usually occupied by constitutive heterochromatin marks disrupts the 3D genome compartmentalisation. This disturbance, in turn, triggers novel chromatin interactions between genic and transposable element (TE) regions. Interestingly, our results imply that epigenetic features, constrained by genetic factors, intricately mold the landscape of 3D genome organisation. This study sheds light on the profound genetic-epigenetic interplay that underlies the regulation of gene expression within the intricate framework of the 3D genome. Our findings highlight the complexity of the relationships between genetic determinants and epigenetic features in shaping the dynamic configuration of the 3D genome.

## Graphical Abstract

**Graphical Abstract:**
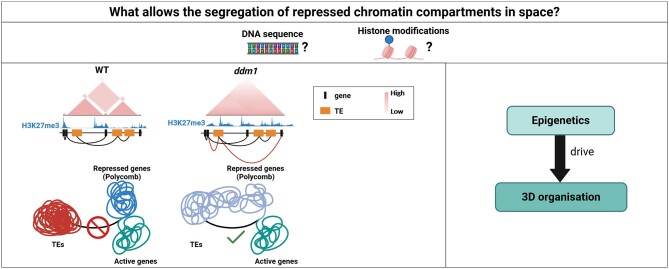

## Introduction

Over time, the traditional perspective of the genome as a linear structure has evolved. Currently, genome folding is studied as a dynamic and intricate 3D chromatin organization process within the nucleus. This 3D genome organization is characterized by multiple hierarchical layers, ranging from chromatin to chromosome territories. In eukaryotes, DNA is wrapped around histone octamers forming nucleosomes that can aggregate each other, creating nucleosome clutches (1). These structures fold again to form domains, with chromatin nanodomains and topologically associated domains (TADs). Widely described in mammals, the latter corresponds to genomic regions that preferentially form contacts with each other rather than the surroundings (2). TADs formation relies on chromatin extrusion, allowed by cohesin complexes, and stops when the chromatin binds to two convergent CTCF proteins, then forming TAD boundaries (3). However, these domains are differently structured in plants: plant genomes lack CTCF homologues and the inside of their TAD-like structures contains mostly transposable elements (TEs) flanked by active regions, while animals’ TADs loop extrusion model tends to bring together distal regulatory sequences with promoter regions or isolates them in space from promoters, and could compete with the local segregation of active and repressed regions (4–6). On a larger scale, TADs associate themselves to form spatially segregated compartments according to their composition. Active compartment (A compartment) corresponds to active chromatin and is associated with active histone marks, genes and active transcription, while B compartment represents inactive chromatin and is defined by repressive epigenetic features from repressive histone marks to DNA methylation and a high density of TEs. The genome displays a pattern of alternating A and B compartments, where A compartments are mainly found in telomeric regions, while B compartments dominate in pericentromeric regions across different crop species (7,8). Therefore, a dynamic spatial segregation orchestrates an interplay between active and repressed regions which ensure the transcriptional coherence of the genome.

Within this intricate 3D organization of the genome, a distinction emerges between two types of chromatin defined as euchromatin and heterochromatin, which primarily differ in their level of condensation and gene activity. Euchromatin is less condensed and transcriptionally active, while heterochromatin is more compact and repressive. Under conventional optical microscopy, heterochromatin exhibits a visually striking and intricately compacted DNA structure, which at the beginning was thought to constitute inactive chromosomal regions (9). Nowadays, it has come to our knowledge that heterochromatic regions play a pivotal role in genome organization and in consequence, influencing the control of gene expression (10–14). Heterochromatin is also known to segregate in two architectural categories, named facultative and constitutive heterochromatin (15,16). These two states are modulated by histone modifications in the N-terminal tails and the presence of other epigenetic features. They are broadly conserved among a wide range of eukaryotic organisms, although their exact definitions may be challenged by some exceptions among organisms (17,18). Overall, facultative heterochromatin refers to a cell type-dependent state characterized by dynamically switching from active to inactive genomic regions, which in plants is generally associated to histone H3 lysine 27 tri-methylation (H3K27me3) and regulated by Polycomb repressive complexes 1 and 2 (PRC1 and PRC2), playing a pivotal role in governing its flexibility (19,20). Conversely, constitutive heterochromatin comprises inactive chromatin regions that remain relatively constant across different cell types within an organism. Characterized by a low gene density, constitutive heterochromatin is associated with DNA methylation, serving as a repressive marker in transcription, particularly in TEs. In the plant model *Arabidopsis thaliana*, *de novo* DNA methylation is instigated by DOMAINS REARRANGED METHYLTRANSFERASE 1 (DRM1) and DRM2 on any cytosine in CG, CHG and CHH (H = A, T or C) context (21). The maintenance of this epigenetic feature involves the RNA-directed DNA methylation (RdDM) mechanisms, encompassing small interfering RNA (siRNA), alongside the action of various methyltransferases depending on the cytosine context (22). For instance, CHG methylation maintenance involves the coordinated action of CHROMOMETHYLTRANSFERASE 3 (CMT3) and several SET domain H3K9 histone methyltransferases, notably KRYPTONITE (KYP) responsible of the deposition of the histone H3 lysine 9 di-methylation (H3K9me2). Both of these enzymes reciprocally maintain themselves through a self-reinforcing loop, in addition to the acknowledged TEs-silencing role of this histone mark (20,23). Beyond these mechanisms, chromatin remodelers like Decreased DNA Methylation (DDM1) also play a crucial role on DNA methylation by providing access to enzymes responsible for its deposition, and particularly targets TEs, as well as DNA wrapped in nucleosomes (24,25). Loss of DDM1 function results in an affected heterochromatin distribution in rice, maize and tomato (26,27). Indeed, tomato encodes two DDM1 genes, *Slddm1a* and *Slddm1b*, and the knock-out of both of these genes leads to an hypomethylation of the gene-poor region transposons in both CG and CHG methylation contexts, and an hypermethylation in the CHH context (28).

All of these distinct chromatin types exhibit spatial segregation within the nucleus, with euchromatin which occupies the centre of the nucleus, facultative heterochromatin which tends to reside in small gatherings within the nucleus, and constitutive heterochromatin, confined to peripheral regions. This segregation appears to be widely conserved, as it is found in both animals and plants (6,29,30), and underscores the fact that some regions with the same repressive transcriptional state are spatially separated. The underlying reason for this spatial segregation of repressed zones remains unclear, prompting questions about whether it could be associated with their sequences or the histone modifications specific to these respective regions. In order to provide answers, we opted for a genetic approach by working with the *ddm1* mutant in tomato. While DDM1 mutation's influence on constitutive heterochromatin through DNA methylation is noteworthy, our primary focus lies in the redistribution of H3K27me3 from gene-rich regions to TEs-rich regions observed in a *ddm1* mutant in *Arabidopsis* (31). With the redistribution of this mark, this mutant provides a valuable avenue for deciphering the potential role of the epigenome in orchestrating the segregation of repressed regions of the genome in space.

Our study aims to elucidate the intricate relationship between spatial segregation of repressed regions and the underlying epigenetic landscape. Therefore, our results unveiled a profound reprogramming of the epigenome induced by DDM1 mutation, marked by a notable relocation of H3K27me3 from gene-rich regions to gene-poor regions, with consequential effects on the transcriptome. Notably, the mutant exhibited a disturbed compartmentalization, suggesting a link between chromatin architecture disruption and the observed epigenome reprogramming. Intriguingly, new interactions emerged in the mutant bridging TEs and genic regions, which highlights the intricate interplay between epigenetic features and 3D organization. Here, we demonstrate that the segregation of repressed regions in space appears to be largely induced by what decorates chromatin, which primarily depends on the sequence of its components.

## Materials and methods

### Plant material and growth conditions

*Solanum lycopersicum* cv. M82 seeds were used in this study. *Slddm1a/Slddm1b* mutant seeds were obtained from Dr Bouché’s laboratory (28). Seeds were germinated on soil and plants were grown in growth chambers at 24°C with a 16/8 h light and dark period. The fourth leaf of 1-month-old plants was used for all experiments in this research.

### *In situ* Hi-C assay

In situ Hi-C experiments were performed according to Concia *et al.* (6) using DpnII enzyme (New England Biolabs). An additional second crosslinking step was performed involving disuccinimidyl glutarate and a second restriction enzyme with DdeI (New England Biolabs) according to Lafontaine *et al.* (32). DNA libraries were prepared using NEBNext Ultra II DNA library preparation kit (NEB) according to the manufacturer's instructions (10 cycles for the PCR amplification step). DNA libraries were checked for quality and quantified using a 2100 Bioanalyzer (Agilent) and the libraries were subjected to 2 × 75 bp high-throughput sequencing by NextSeq 500 (Illumina). Three independent biological replicates were generated.

### Analysis of Hi-C data

Raw reads from FASTQ files were pre-processed with Trimmomatic-0.38 to remove Illumina sequencing adaptors. -5′ and -3′ ends with a quality score <5 was trimmed and reads <30 bp after trimming were dropped. The cleaned reads were processed with HiC-Pro 3.1.0. The reads were aligned using Bowtie2.4.4 onto the M82 V1.0 genome assembly (33) with very-sensitive mode. Invalid ligation products (such as dangling ends, fragments ligated on themselves and ligations of juxtaposed fragments) were discarded. Valid pairs were used to produce raw interaction matrixes at different resolutions. After, ‘.hic’ files were generated with the hicpro2juicebox.sh script which belong to HiC-Pro and visualized with the tool Juicebox 1.11.08. Hi-C chromatin interaction heatmaps were visualised using cooltools (https://github.com/open2c/cooltools) at 100 and 50 kb resolutions. The genome-wide insulation score and aggregate peak analysis were performed by GENOVA v1.0.1. We used Pentad to calculate and visualize quantitative interactions in different compartments. The function hicAggregateContacts V3.7.2 which originated from HiCExplorer (34) was used to plot aggregated Hi-C sub-matrices of a specified list of positions. The Epigenome Browser WASHU was used to display the interaction loops called from Homer.

### ChIP-seq assay

ChIP-seq assays were performed according to Ramirez-Prado *et al.* (35) with extra modifications to the chromatin extraction step cross-linked plants were ground using gentleMACS (Miltenyi Biotec) with the 4C program in an extraction buffer (50 mM HEPES–KOH pH 7.5, 150 mM NaCl, 1 mM EDTA, 1% Triton X-100, 0.1% sodium deoxycholate, 1% SDS) containing protease inhibitors. The mixture was filtered through a 50 μm filter and left on ice for 10 min. After centrifuge for 10 min at 1500g at 4°C, the supernatant was removed and the pellet was resuspended in sonication buffer (50 mM HEPES KOH pH 7.5, 150 mM NaCl, 1 mM EDTA, 1% Triton X-100, 0.1% SDS) containing protease inhibitors. 4 μg of anti-H3K9me2 (Abcam, ab1220), anti-H3K27me1 (Millipore, 07-448), anti-H3K27me3 (Millipore, 07-449) and anti-H3K9ac (Millipore 06-942) antibodies were used in an antibody incubation buffer with a final SDS concentration of 0.1%. ChIP-seq libraries were prepared from 10 ng of DNA using NEBNext Ultra II DNA Library Prep Kit for Illumina (NEB) according to the manufacturer's instructions. Two independent biological replicates were generated for each genotype. DNA libraries were checked for quality and quantified using a 2100 Bioanalyzer (Agilent), size-selected for 200–500 bp fragments using PippinHT (Sage Science) and subjected to 1 × 75 bp high-throughput sequencing by NextSeq 500 (Illumina).

### Analysis of ChIP-seq data

Trimmomatic-0.38 was used for trimming with the following parameters: minimum length of 30 bp; mean Phred quality score >30; leading and trailing bases removal with base quality <5. The reads were mapped onto the M82 V1.0 genome assembly using Bowtie2.4.4 with mismatch permission of 1 bp. To identify significantly enriched regions, we used MACS V3.0.0a6 with the default peak-calling parameters except broad peak (H3K27me3), *q*-value threshold:0.05, extsize 150. To extract the average scores across the genomic regions, multiBigwigSummary command from the deepTools package was used with default parameters on the S3norm normalised bigWig files (36). The heatmap and peak profiles were generated by deepTools. Mapped BAM files were converted to bigWig format using deepTools and S3norm normalised to configure the tracks in JBrowse2.

### Immunostaining

Tomato leaves were fixed by vacuum infiltration for 20 min with 1× PBS–3% Formalin, and washed five times with 1× PBS. Nuclei were extracted by cutting the leaves with a razor blade in a solution of 1× PBS–0.1% supplemented with Triton X-100 (w/v), filtered on a 50 μm filter and centrifuged at 600g for 8 min at 4°C. The supernatant was carefully removed and the pellet was washed 3 times with 1× PBS–0.1% Triton X-100, and one more wash with 1× PBS only. After a centrifugation with above parameters, the pellet was resuspended with 1× PBS and a drop of the mixture was deposited on a poly-lysine slide and air-dried for 30 min at 37°C. Slides were rehydrated with PBS and washed 5 times with PBS-0.1% Tween and incubated 30 min at RT with 1× PBS–0.1% Tween–3% BSA. Slides were placed in a moist chamber and incubated overnight at 4°C with primary antibodies (400X diluted) of H3K9me2 (Active Motif, 39753), H3K27me3 (Active Motif 61017), H3K9ac (Active Motif 39918), H3K27me1 (Millipore 07-448). Slides were washed and then incubated for 1h at RT in the dark with Goat anti-Rabbit Alexa Fluor Plus 488 (A11034 Invitrogen) and Goat anti-Mouse Alexa Fluor Plus 555 (A32723 Invitrogen) secondary antibodies (400× diluted). DNA was counterstained with 4,6 diamidino-2-phenylindole (DAPI) in Vectashield Diamond Antifade mounting media. Finally, slides were imaged on a confocal microscope (Zeiss Microsystems, and Leica Microsystems).

### RNA-seq assays and libraries preparation

Total RNA was extracted from the fourth leaves of 1-month-old plants with Nucleospin RNA kit (Macherey-Nagel), according to the manufacturer's instructions. RNA-seq libraries were prepared from 2 μg of total RNA using NEBNext Ultra II Directional RNA library Preparation Kit (NEB) according to the manufacturer's instructions for the poly(A) mRNA workflow. Total RNA-seq libraries were prepared from 500 ng of total RNA using Zymo-Seq RiboFree Total RNA Library Kit. Both libraries were checked for quality and quantified using a 2100 Bioanalyzer (Agilent) and subjected to 1 × 75 bp high-throughput sequencing by NextSeq 500 (Illumina). Two independent biological replicates were generated.

### Differential expression analysis

Single-end reads of RNA-seq samples were trimmed using Trimmomatic-0.38 with the parameters: minimum length of 30 bp; mean Phred quality score >30; leading and trailing base removal with base quality <5. Hisat2.2.1 aligner was used to map the reads to the M82 V1.0 genome assembly. Raw read counts were then extracted using the featureCounts v2.0.0 utility from the Subread based on the M82 v1.1.1 gene annotations. Afterward, we used DESeq2 v1.34.0 to identify differentially expressed genes (DEGs). R package Rideogram V0.2.2, gridExtra V2.3 and ggplot2 V3.4.2 were used to produce the scatterplots for differentially expressed genes. KOBAS (37) and clusterProfiler (38) is performed for gene ontology terms analysis, and we used ggplot2 V3.4.2 to visualise the GO results. To quantify the TE expression, star2.7.10b aligner was used to map the clean reads to the M82 V1.0 genome with the following parameters: –sjdbScore 2, –winAnchorMultimapNmax 100 and –outFilterMultimapNmax 100. Then, we used Tetranscripts V2.2.3 (39) to count reads mapping on TEs (40) and perform DESeq2 v1.34.0 to differential expression analyses in R environment.

## Results

### *ddm1* exhibits a major redistribution of constitutive and facultative heterochromatin marks

**Figure 1.**
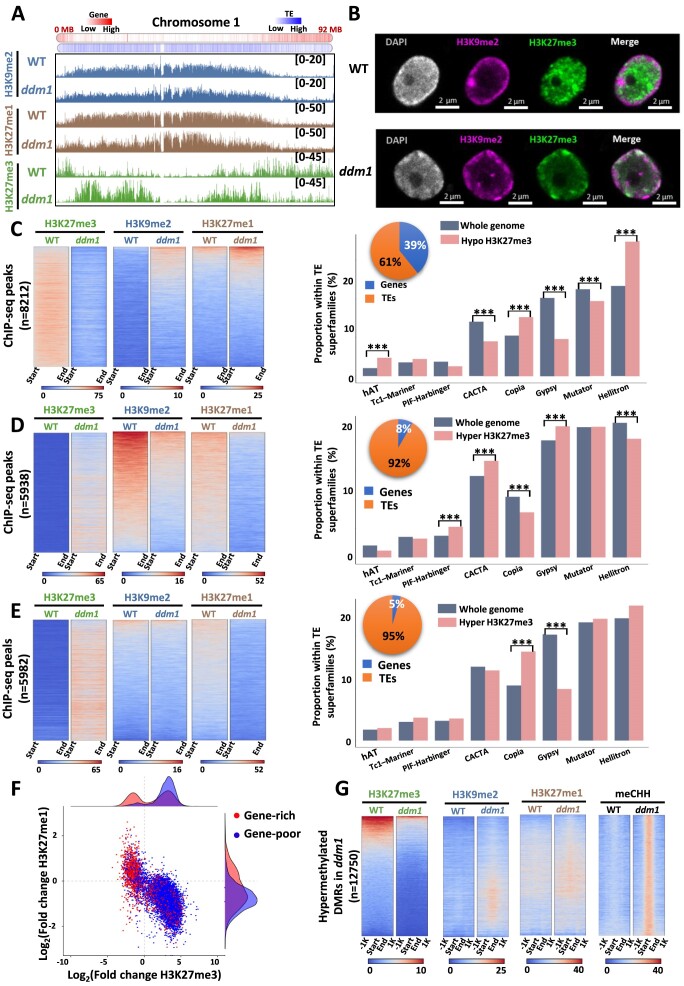
*ddm1* displays a reconfiguration of its epigenome. (**A**) Screenshot of chromosome 1 from JBrowse2 genome browser representing H3K9me2 (blue), H3K27me1 (brown) and H3K27me3 (green) ChIP-seq data in wild type and *ddm1* mutant. Gene density is displayed at the top with a higher density of TEs and genes respectively in blue and red. (**B**) Immunofluorescence detection of H3K9me2 (pink) and H3K27me3 (green) histone modifications and DAPI staining (grey) in isolated tomato nucleus from wild type and *ddm1*. (C–E) On the left panels, heat-maps representing ChIP-seq profiles for H3K27me3 (green), H3K9me2 (blue), and H3K27me1 (brown) within H3K27me3 hypomethylated regions in *ddm1* compared to the wild type (**C**); within H3K27me3 hypermethylated regions and H3K9me2 hypomethylated regions (**D**); within H3K27me3 hypermethylated regions and no change in H3K9me2 regions (**E**). The color gradient from blue to red characterize the counts from low to high in the respective regions. On the right panels, TEs superfamilies repartition in percentage for the whole tomato genome (grey) and for regions of each class (pink). Pie charts represent the proportion of genes (blue) and TEs (orange) in each class. *** corresponds to significant differences (pvalue < 5% with a Student test) observed between two groups indicated by black bars. (**F**) Scatter plot of TEs in gene-rich regions (red), TEs in gene-poor regions (blue) with trend line according to the fold change of H3K27me3 on x-axis and H3K27me1 on y-axis. (**G**) Heatmaps of ChIP-seq signals (H3K27me3 (green), H3K9me2 (blue), H3K27me1 (brown)) and DNA methylation (CHH (black)) from differentially methylated regions (DMRs) in *ddm1* compared to WT and selected in Corem *et al.* (2018).

To investigate the relationship between genetics and epigenetics in shaping the 3D genome structure, we characterized the epigenetic landscape of the tomato *Slddm1a/Slddm1b* double mutant (*ddm1*), which was shown to display severe modifications of DNA methylation both in heterochromatic and euchromatic regions (28). We performed ChIP-seq analyses in wild type (WT) vs *ddm1* of two post-translational histone modifications marks associated with constitutive heterochromatin, H3K9me2 and H3K27me1, and the Polycomb-dependent facultative heterochromatin-related mark H3K27me3 (Supplementary Table S1). At the chromosomal level, a notable finding was the relocation of H3K27me3 from regions rich in genes to gene-poor areas (Figure 1A). Although the deposition of H3K9me2 and H3K27me1 was also impacted in *ddm1*, their redistribution did not occur at the scale observed for H3K27me3. To confirm these changes, we performed immuno-localization assays for several histone modifications on isolated nuclei from WT and *ddm1* (Figure 1B and Supplementary Figure S1). Interestingly, a strong H3K27me3 signal was observed at the periphery of *ddm1* nuclei compared to its central localization in the WT, confirming the relocation of this mark observed through ChiP-seq data analyses (Figure 1B). A deep analysis of ChIP-seq data revealed three distinctive patterns of H3K27me3 redistribution: one marked by a loss of H3K27me3 and either a gain or no changes on both H3K9me2/H3K27me1, another with a H3K27me3 gain and H3K9me2/H3K27me1 losses, and a third with an H3K27me3 increase, a reduced H3K27me1 and a stable but low level of H3K9me2 (Figure 1C, D and E, respectively), suggesting that the gain of H3K27me3 could occur only on regions displaying either a low level or a decrease of H3K9me2. Peak annotation indicated that the first class, which is associated with a loss of H3K27me3 in *ddm1*, displayed a higher proportion of genes compared to other classes (39%) (Figure 1C). In contrast, the other two classes which are associated with a gain of H3K27me3 in *ddm1* displayed a high proportion of TE (92–95%) (Figure 1D and E). With a significant portion of the epigenomic alterations taking place on TEs within each cluster, our exploration delved into their respective associated superfamilies. Our findings revealed connections between at least eight superfamilies and these three distinctive chromatin signatures. Notably, the overall involvement of TE superfamilies differs across the three clusters. For instance, while the LTR/COPIA and LTR/Gypsy superfamilies were identified in all clusters, they exhibited varying degrees of contribution within each cluster (Figure 1C–E). Therefore, considering both the role of DNA methylation in TEs repression and our current results, we explored the relationship between their location and their chromatin status. For that, we classified TEs in two groups: (i) TEs present in gene rich regions and (ii) TEs present in gene poor regions and analysed their epigenetic status. We observed that TEs present in gene poor regions are enriched in H3K27me3 and depleted in H3K27me1 and H3K9me2 (Figure 1F). On the other hand, approximatively one half of TEs present in gene rich regions showed a gain in H3K27me3 accompanied by a loss of H3K27me1 or H3K9me2, whereas the other half displayed a loss in H3K27me3 with a gain in one of the other two histone marks, exhibiting a position-dependent asymmetry in the redistribution of histone marks (Figure 1F, Supplementary Figure S2). It was previously reported that the *ddm1* mutation leads to a global loss of DNA methylation, with a redistribution of methylation in the CHH context at heterochromatic TEs (28). Therefore, we hypothesized a potential relationship between the redistribution of histone marks and DNA methylation. To this end, we integrated differentially methylated regions (DMRs) gaining either CG, CHG or CHH methylation and our ChIP-seq data. Our findings revealed that regions gaining DNA methylation in *ddm1* were associated either with a decrease in H3K27me3, accompanied by minimal alterations in H3K9me2 and H3K27me1 or with regions initially devoid of H3K27me3, yet exhibiting a slight increase in H3K9me2 and H3K27me1 (Figure 1G and Supplementary Figure S3). Our results suggest that there is an anti-correlation between DNA methylation and the redistribution of H3K27me3 in *ddm1*. Overall, our findings indicate that *ddm1* mutation induces a massive epigenome reconfiguration characterized by a redistribution of facultative heterochromatin marks in regions usually occupied by constitutive heterochromatin marks.

### Epigenome reconfiguration correlates with transcriptome reprogramming

**Figure 2.**
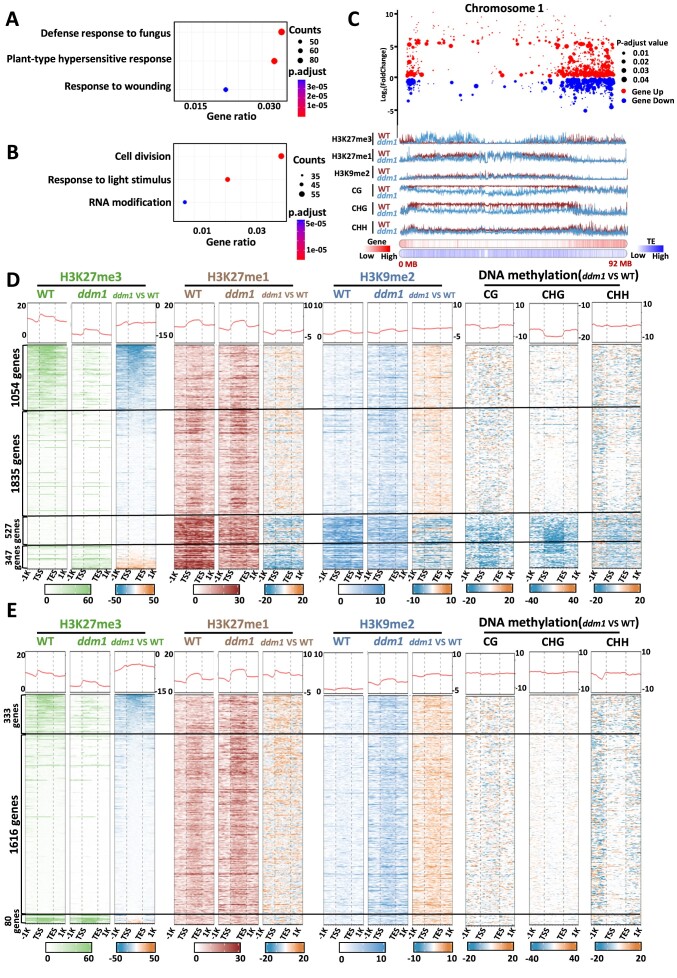
The reconfiguration of ddm1 mutant epigenome impacts its transcriptome. (**A**) Gene ontology enrichment of up-regulated genes. (**B**) Gene ontology enrichment of down-regulated genes. Counts correspond to the number of genes associated with the corresponding GO enrichment term. *P*.adj were calculated using Fisher's exact test. (**C**) Scatter plot of differentially expressed genes in chromosome 1. Up-regulated (red) and down-regulated genes (blue) with associated and adjusted *P*-value. ChIP-seq data (H3K27me3, H3K27me1 and H3K9me2), DNA methylation data (CG, CHG and CHH), gene and TE density are linked with the plot at the bottom of the subfigure, wild type (brown) and *ddm1* (light blue). (D, E) Heatmaps of up-regulated (**D**) and down-regulated (**E**) genes in the wild type and *ddm1*. H3K27me3 (green), H3K27me1 (brown), H3K9me2 (blue); and DNA methylation data in all contexts (black). Each histone mark has a heatmap (right) showing the difference between *ddm1* and the wild type. All DNA methylation heatmaps are represented as differences between *ddm1* and WT. Each row corresponds to one gene and were ranked according to the H3K27me3 signal.

In order to assess the effect of the epigenome reconfiguration on transcriptome reprogramming, we performed RNA-seq analyses in *ddm1* (Supplementary Figure S4). We found nearly 5800 differentially expressed genes (DEGs) in *ddm*1, of which 3763 were up-regulated and 2029 down-regulated (Supplementary Table S2). Gene ontology analysis (GO) revealed that up-regulated genes were enriched in GO terms involved in response to biotic stress, whereas an enrichment for genes involved in cell division, response to light stimulus and RNA modification was observed for the set of down-regulated genes (Figure 2A, B). Subsequently, we integrated ChIP-seq and RNA-seq data to visualize the global changes in gene expression and repartition of H3K9me2, H3K27me1 and H3K27me3. The visual representation illustrating the distribution of histone marks and gene expression patterns along chromosome 1 indicated massive changes in gene expression and fluctuations in these three histone marks in both gene-dense and TE-rich regions in *ddm1* (Figure 2C, Supplementary Figure S5). Thus, we aimed to determine the epigenomic profiles associated with up- and down-regulated genes. As *ddm1* participates in DNA methylation, we integrated publicly available datasets (28) to our analyses. Among the 3763 up-regulated genes, 76.8% (*n* = 2889) showed no alterations in DNA methylation levels in *ddm1* compared to WT, associated with modest gains in both H3K27me1 and H3K9me2 (Figure 2D, Supplementary Figure S6). In this subset, 28% (*n* = 1054) displayed a loss of H3K27me3, suggesting that those genes are directly controlled by Polycomb Repressive Complexes (PRCs). The remaining 48.8% (*n* = 1835) exhibiting a minimal level of H3K27me3 did not show any changes of this chromatin mark, indicating the independence of these genes from active PRC regulation. 14% (*n* = 527) of the up-regulated genes exhibited a loss of DNA methylation in all contexts, along with a loss of both H3K27me1 and H3K9me2 and no changes on H3K27me3. The remaining 9.2% (*n* = 347) showed a loss of both H3K27me1 and H3K9me2 and a gain in H3K27me3, which suggests that H3K27me3 is not sufficient to repress this group of genes. In contrast, all down-regulated genes showed little to no changes in DNA methylation levels, along with H3K9me2 and H3K27me1 gain (Figure 2E, Supplementary Figure S6). H3K27me3 levels seemed to have little role in their down regulation, since among them, the majority 79.6% (*n* = 1616) did not present any changes of H3K27me3, 16.4% (*n* = 333) exhibited a loss of H3K27me3, and only 3% (*n* = 80) showed a very modest gain of H3K27me3, suggesting that their repression is probably due to an increase of the deposition of H3K9me2 that appears to be independent of DNA methylation. Furthermore, we conducted the same analyses on deregulated TEs. Overall, up-regulated TEs seems characterized by a gain in H3K27me3 and a loss of DNA methylation and constitutive heterochromatin marks, whereas down-regulated TEs display a less straightforward depiction (Supplementary Figures S7 and S8; Supplementary Table S3). It was proposed that H3K27me3 could function as a back-up mechanism for hypomethylated TEs in mammals and *Arabidopsis thaliana* (41,42). To investigate whether this mechanism operates similarly in tomato, we divided all up-regulated TEs into two groups: up-regulated TEs that gained H3K27me3 in *ddm1*, and the remaining up-regulated TEs. We then compared their expression levels. Interestingly, the up-regulated TEs that gained H3K27me3 exhibited significantly lower expression levels compared to those without this histone modification. This suggests that H3K27me3 may act as a back-up mechanism for hypomethylated TEs in tomato (Supplementary Figure S9). Altogether, our results revealed that a major epigenome reconfiguration occurs in *ddm1* and leads to a transcriptome reprogramming.

### Genome compartmentalization is affected in *ddm1*

**Figure 3.**
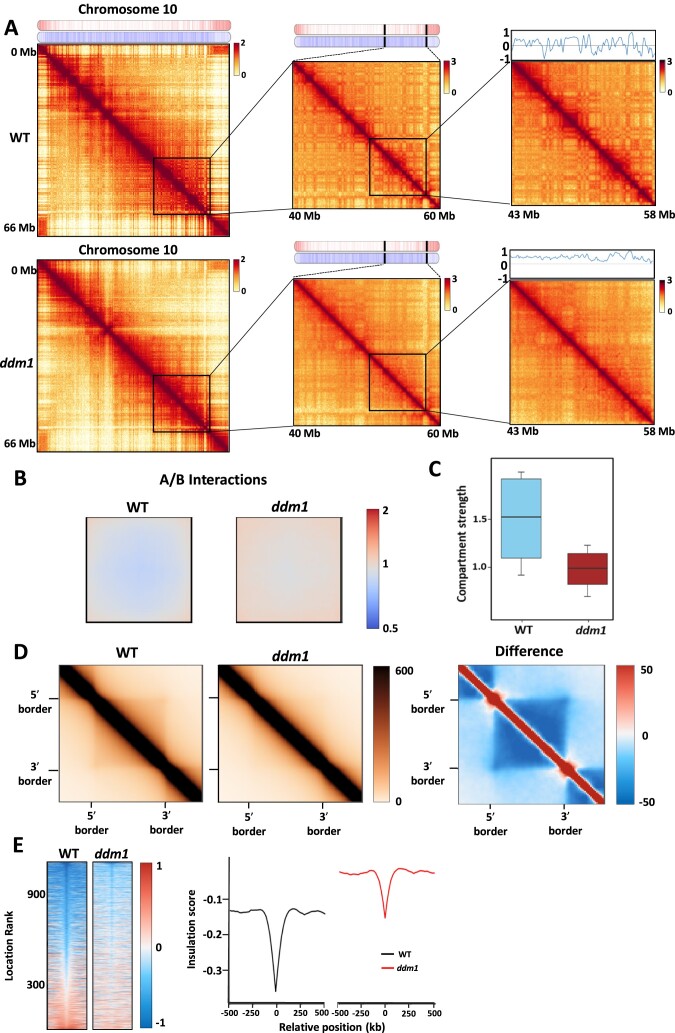
Chromatin architecture is affected in the ddm1 mutant with a weakened compartmentalization. (**A**) Chromosome 10 Hi-C interaction map at increasing levels of resolution. Gene and TE density are pictured at the top of the first map and a quantification of the compartmentalization, represented by a binary segmentation of the eigenvector (obtained with cooltools software) is shown on the top of the last map. (**B**) Analysis of compartment dynamics according to the interactions between compartments for the wild type and *ddm1* (obtained with Pentad software). (**C**) Boxplot of compartment strength calculated from inter- and intra-compartment interactions for the wild type and *ddm1*. (**D**) Aggregate TAD analysis in wild type and *ddm1* (left), and differential analysis is presented (right). Dark blue denotes a loss of interaction in *ddm1* (obtained with GENOVA software). (**E**) Insulation heatmap (left) with a main insulation profile represented as the insulation score versus the relative position in kb (right) in wild type (grey) and *ddm1* (red). The right panel shows an average score and each row corresponds to a TAD-border in the left panel.

Based on the major redistribution observed between constitutive and facultative heterochromatin marks, our study suggested that *ddm1* mutant could serve as a suitable model for gaining a deeper understanding of the role of the epigenome in 3D chromatin organisation. Therefore, we evaluated the *ddm1* genome architecture by performing Hi-C analyses (Supplementary Figure S10). The analysis through Hi-C intra-chromosomal contact maps unveiled a disparity in the strength of interactions between distant domains and compartmental segregation in *ddm1* compared to the WT, as illustrated for chromosome 10 (Figure 3A, Supplementary Figure S11). This difference implies significant alterations in the 3D genome structure. To validate this observation, we calculated PC1 values from Hi-C data, where positive and negative values were indicative of the discrimination between active (compartment A) and inactive (compartment B) compartments. As illustrated in the zoomed-in Hi-C map, the line chart representing PC1 values displayed a comparatively more uniform trend in *ddm1* as opposed to the WT, indicating a defect in chromosome compartmentalization in the *ddm1* background (Figure 3A, Supplementary Figure S11). To go further, we first quantified the genome-wide compartmentalization by computing the average compartments A and B, and visualizing intercompartmental interactions in *ddm1* in comparison to the WT. Notably, we observed heightened interactions between A and B compartments in *ddm1* (Figure 3B, Supplementary Figure S11). To confirm the weakened segregation observed between compartments in *ddm1*, we used a second method to measure compartment strength, defined as the number of intracompartment interactions (AA or BB) compared to the number of intercompartment interactions (AB). Our analysis revealed that the increased intercompartment interactions corresponded to a reduction in intracompartment interactions, ultimately leading to a weakened compartmentalization in *ddm1* (Figure 3C, Supplementary Figure S11). Given that TAD-like structures in plants typically consist of extensive repressed chromatin domains flanked by active regions (7), we postulated that if compartmentalization is compromised, TAD-like structures would similarly be impacted in the *ddm1* context. Hence, we generated aggregated plots showing the average of all TAD-like structures in the WT and *ddm1*. In addition, to obtain a clear distinction in the structural changes between *ddm1* and the WT, we calculated the difference between the two plots revealing a reduction in interactions inside the TAD-like structure in *ddm1* (Figure 3D, Supplementary Figure S11). Our analyses implied that an increased number of interactions outside the TAD-like structures could occur, leading to weakened TAD-like boundaries in *ddm1* in contrast to the WT. To verify this prospect, we generated heat maps showing the genome-wide insulation score at TAD-like borders, along with the mean insulation profiles. We observed that in addition to a higher average insulation score in *ddm1* compared to the WT, the average signal drops less than the WT one at the TAD-like border (Figure 3E, Supplementary Figure S11). Altogether, our results showed that *ddm1* exhibits weakened compartmentalization, linked to weakened TAD-like borders, potentially attributed to impaired segregation between genic and TEs regions.

### Epigenome reconfiguration impacts the 3D genome organization

**Figure 4.**
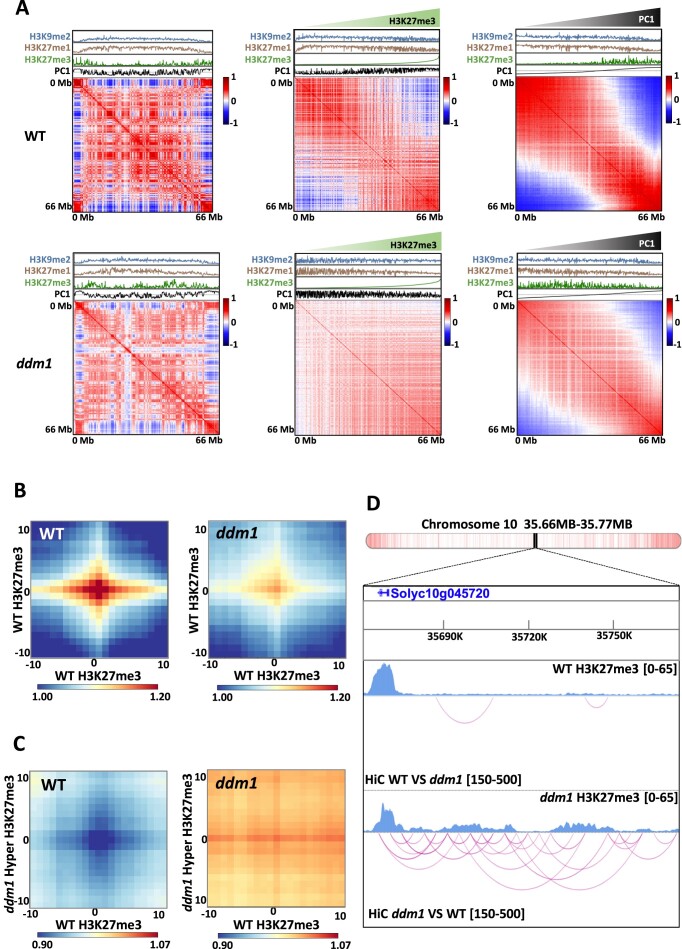
Reconfiguration of the epigenome in ddm1 mutant induces new interactions across the genome. (**A**) Pearson correlations of distance-normalized Hi-C interaction frequency maps of chromosome 10. The ChIP-seq signal for H3K9me2 (blue), H3K27me1 (brown), H3K27me3 (green) and PC1 component from principal component analysis (black) were aligned to the map, for wild type and *ddm1* (left). Maps with reorganized bins according to their H3K27me3 (middle) and PC1 value (right). (B-C) Aggregate plots quantifying the mean of aggregated WT and *ddm1* Hi-C contacts matrices between regions marked with H3K27me3 in the wild type (**B**) and between new regions marked with H3K27me3 in *ddm1* versus the regions previously mentioned in the wild type (**C**). (**D**) Screenshot of a region in the chromosome 10 from WashU genome browser for H3K27me3 ChIPseq data, depicted in light blue, and Hi-C differential interactions arcs (pink). Differential interaction arc intervals were chosen to illustrate the most significant Zscores corresponding to WT-specific interactions at the top, and *ddm1*-specific interactions at the bottom. Gene density (with white corresponding to a low density and red, a high density of genes) of the chromosome and the portion of the region can be seen at the top of the screenshot.

**Figure 5.**
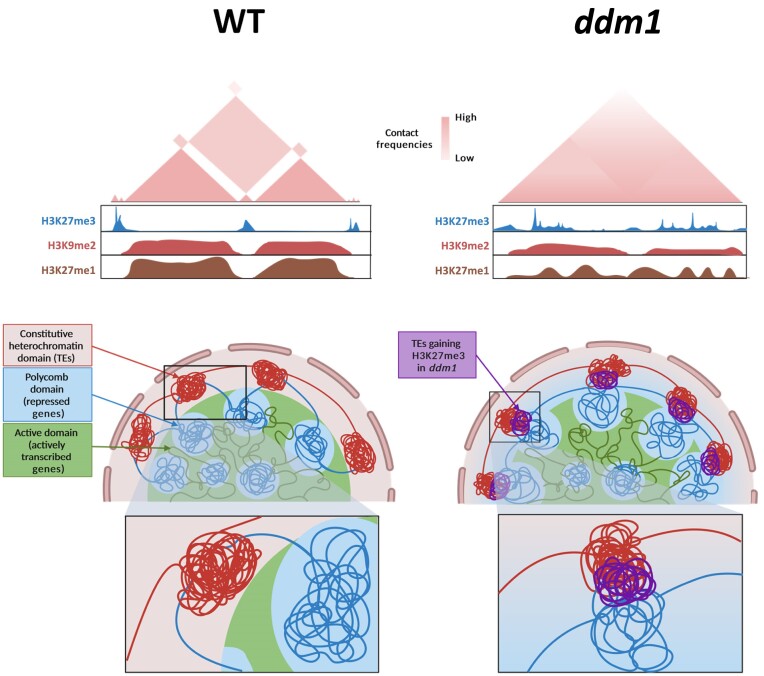
Model summarizing how epigenetic modifications orchestrate the interplay between genic and TEs regions. There is a clear segregation between the different chromatin constituents in the wild type: constitutive heterochromatin domains (red), Polycomb domains (blue) and active domains (green). The distribution of H3K27me3 (blue), H3K9me2 (red) and H3K27me1 (brown) marks coincides with the establishment of separate compartments. In contrast, the compartmental delineation is considerably more diffuse in *ddm1*. The ectopic depositions of H3K27me3 and the general reshuffling of the other histone marks induce new interactions, particularly between genic and TEs regions (violet). Created with Biorender.

To elucidate the connection between the disruption of compartmentalization and the epigenomic alterations in *ddm1*, we integrated our Hi-C data with ChIP-seq data. First, we computed the Pearson correlation of the distance-normalized interaction maps. As anticipated, the maps for *ddm1* exhibited notable distinctions from those of the WT (Figure 4A, Supplementary Figure S12). Due to this significant dissimilarity, we sorted our Hi-C maps according to different features, including H3K27me3 and PC1 values. To this end, we rearranged the rows and columns of the correlation matrix by sorting the bins based on the ascending signal of the selected modality. In the maps sorted by H3K27me3, we observed compartmentalization in the WT, allowing the segregation between regions strongly marked with H3K27me3 and regions marked with constitutive-related marks. In contrast, in *ddm1* this segregation was not visible, highlighting the potential role of histone marks in driving 3D organization. To support this, maps sorted by PC1 showed segregation between compartments and indicated a clear segregation between regions marked with constitutive and with facultative heterochromatin related histone marks in the WT, which was not as obvious in *ddm1* (Figure 4A, Supplementary Figure S12). We performed the same analysis for all three DNA methylation contexts. As expected, when sorted according to CG, CHG and CHH values, the matrix displayed a clear segregation into two types of compartmental domains. In addition, similar to the H3K27me3 sorted map, this clear compartmentalization is not observed in *ddm1* tomato mutant. These results highlight that DNA methylation strongly correlate with compartmental organization in tomato. (Supplementary Figure S13). These observations suggested that new interactions between genic and TEs-rich regions result in *ddm1* from a comprehensive epigenome reprogramming, and hinting at the histone mark H3K27me3 as a pivotal feature for the formation of new contacts in *ddm1*. To test this hypothesis, we generated aggregate plots (aggregated Hi-C matrices) to quantify the mean of aggregated Hi-C submatrices between regions marked by H3K27me3 in the WT, following an observed/expected transformation of the Hi-C matrix for both WT and *ddm1*. We observed that those loci marked by H3K27me3 in the WT interact with each other in the WT and less intensely in *ddm1* (Figure 4B). Secondly, we generated aggregate plots to assess the strength of contacts between loci already designated by H3K27me3 in the WT and loci solely marked by H3K27me3 in *ddm1*. Interestingly, this analysis unveiled an increase in contact strength in *ddm1* compared to the WT, particularly between loci marked by H3K27me3 only in *ddm1* and those already marked by H3K27me3 in the WT (Figure 4C, D and Supplementary Figure S14). To determine if new interactions in *ddm1* could be associated with active histone mark, we performed a ChIP-seq using H3K9ac antibody. By integrating ChIP-seq and Hi-C in both WT and *ddm1*, we observed that regions showing an increase H3K9ac level in *ddm1* compare to WT displayed new homotypic interactions with euchromatic regions. In addition, we observed a strong correlation between the level of H3K9ac, the transcription and interaction strength, suggesting that the gain of active histone marks may induce the formation of novel interaction that could lead to an increase of gene expression. Therefore, it highlights the potential role of active histone modifications in 3D genome organization (Supplementary Figure S15). Altogether, our research depicts a mechanism where epigenetics shapes the 3D genome organization by separating regions based on their epigenomic features, playing a major role in driving spatial compartmentalization than previously attributed (Figure 5).

## Discussion

In this study, we provide a comprehensive analysis of the tomato epigenome's role in 3D genome structure, with a focus on the influence of the epigenome in spatial segregation of genomic repressed regions. Using the tomato *ddm1 crispr* mutant, impaired in DNA methylation, we discovered a massive epigenome reshaping linked to a redistribution of H3K27me3 from gene-rich regions to TE regions. Furthermore, histone mark redistribution extends to other histone modifications. Thereby, after delving into *ddm1* epigenome reorganization, we unveiled novel interactions between genic regions and TEs regions that possibly lead to the reprogramming of the transcriptome and the 3D genome architecture. Therefore, TEs, which are considered as a major determining factor of DNA methylation patterns limit the distribution of histone marks across the genome, ultimately shaping the 3D organisation of the genetic information in the cell nucleus. Our findings reveal an intricate genetic-epigenetic interplay driving the control of gene expression within the configuration of the 3D genome and chromatin compartmentalization. Akin to the plant model *Arabidopsis thaliana*, which lacks an obvious TAD-like genome packing (43), the tomato genome contains TAD-like domains that overlap with local compartments (7). Our investigation remarkably reveals that this compartmentalization is impaired in *ddm1*, which is directly associated with weakened TAD-like borders.

DNA methylation and H3K27me3 are generally considered as mutually exclusive epigenetic features. Previous studies have shown that PRC2 encounters difficulties when interacting with nucleosomes containing methylated DNA, indicating that DNA methylation may prevent H3K27me3 deposition, even when they can co-exist in low CG density regions (44). Moreover, depletion of DNA methylation results in the scattering of H3K27me3 in regions not targeted by Polycomb (45). In the *ddm1* tomato genome context, we identified an epigenomic landscape distinguished by H3K27me3 gain upon the loss of DNA methylation, as previously observed in *Arabidopsis* plants defective in DDM1 (31). Interestingly, histone mark redistribution extends beyond H3K27me3, affecting the distribution of other histone modifications. The gain of H3K27me3 leads to either the loss of H3K9me2 and H3K27me1, or only the loss of H3K27me1 without affecting the low amount of H3K9me2. These two events are linked mainly to TEs. In addition, our results showed a particular transcriptomic profile associated with *ddm1* epigenome reconfiguration. Our results disclose a strong enrichment of plant defence-related GO terms for overexpressed genes, providing support for previous research that highlights the emerging relevance of H3K27me3 as an important feature for plant immunity regulation (46–48). Interestingly, almost 80% of the upregulated genes and all down-regulated genes showed no changes in already low DNA methylation levels, indicating that histone mark deposition has a strong influence in *ddm1* transcriptome dynamics. Previous research in *Arabidopsis* reported an interdependent loop between the H3K9 histone methyltransferase KYP, and the DNA methyltransferase CMT3. This loop is responsible for the maintenance of DNA methylation in the CHG context (23). We identified a proportion of up-regulated genes gaining H3K9me2. Besides, this epigenetic signature also occurs in the majority of repressed genes. Therefore, we hypothesized that for overexpressed genes, gain of H3K9me2 in *ddm1* could be partially attributed to a disruption of the KYP-CMT interdependent loop. However, previous studies have demonstrated that H3K27me3 is necessary but not sufficient for PcG-mediated silencing (49). Interestingly, we identified a small proportion of up-regulated genes (9.2%) that gain H3K27me3, and lose H3K27me1, H3K9me2, alongside a strong DNA methylation loss in all contexts, suggesting that these genes are actively repressed by DNA methylation, and that H3K27me3 is not sufficient to maintain this repressive state. In mouse somatic cells, the loss of DNA methylation is sufficient to induce de-repression of *Hox* genes, which are repressed by PcG genes (50). Thus, the gain of H3K27me3 in *ddm1* does not uniformly repress all targeted genes and TEs when DNA methylation is lost, suggesting that alternative epigenetic mechanisms control the expression dynamics of this category of genomic elements.

Chromatin dynamics must be considered to understand its influence on temporal and spatial gene expression. Recent advances in chromosome conformation capture experiments have enabled the observation and characterization of distinct components within the chromatin organization, helping to characterize genome configurations among species. In model organisms such as *Drosophila*, a marked correlation was found between chromatin states and genome structure at both compartment and TAD levels. TADs are mostly composed of condensed repressed domains separated by active chromatin regions, which are enriched by internal interactions, while compartments correspond to long-range connections between domains sharing similar epigenetic features (51–53). These findings suggest a preferential folding of genome units, leading to interactions within themselves and with homotypic domains. Mutual exclusion between different chromatin features appears to be sufficient to generate a TAD-like pattern in species like *Drosophila* unlike mammals. TADs primarily align with transcribed regions and encompass a variety of chromatin types. However, their compartments still display the previously stated correspondence between chromatin state and long-range interactions (2,54). Cohesin depletion induces the spontaneous creation of TAD-like domains appearing to form according to their chromatin type, while displaying incoherent boundaries (55,56). The fact that these borders are mainly the result of loops induced by the action of cohesin and CTCF proteins suggests that 3D organization in mammals at the TAD scale requires two competing mechanisms: loop extrusion and compartmentalization defined by epigenetic states (57). Plants can enter in the category in which chromosomal contacts align closely with the epigenome (58,7). In this regard, our findings depict that short- and long-range contacts are affected by ectopic epigenetic states in *ddm1*, triggering increased inter-compartment interactions, thereby disrupting the edges of TAD-like structures. This underscores that genome compartmentalizzation at all scales is epigenome-dependent. Histone modifiers influence chromatin architecture, such as H3K27me3, which plays a crucial role in the generation of short- and long-range chromatin loops in *Arabidopsis* (59). On top of that, our observations related to the H3K27me3 redistribution leading to the establishment of new strong chromatin interactions, unveil the central role of histone marks in orchestrating the 3D genome organization.

The distinctive segregation between euchromatin and heterochromatin is a key mechanism influencing genome folding. However, the specific reasons behind the spatial exclusion between constitutive and facultative heterochromatin remains unclear. Polymer modelling studies suggested that interactions among heterochromatin regions contribute to the phase separation between active and inactive genome regions (13). Di Stefano *et al.* proposed that self-attracting nucleolar organizing regions (NORs) and constitutive heterochromatin participates in chromosome organisation in *Arabidopsis*. Notably, their observations suggest that self-attraction among facultative heterochromatin regions promotes the formation of Polycomb bodies housing gene clusters enriched with H3K27me3 (14), highlighting the prospective role of the epigenome in the segregation of transcriptionally repressed but spatially separated regions. In this regard, our results demonstrate an actual role for epigenetic modifications on the segregation between repressed genic regions and TEs. Indeed, the redistribution of H3K27me3 from gene-rich regions to TE-rich regions in *ddm1* induced new interactions between repressed regions that did not interact in the WT. This leads to the assumption that the separation between regions sharing the same transcriptionally repressed state does not inherently rely on the sequence of those regions, but rather on their epigenetic signature normally characterizing each genomic element. Several reports suggest that DNA sequence restricts the epigenome by limiting the implementation of specific chromatin states in specific regions, thereby directing a distinct distribution of chromatin marks (60–62). Epigenetics could shape the 3D genome organization by separating regions based on their epigenomic features, playing a major role in driving spatial compartmentalization than previously attributed. Future research based on single-cell epigenome analysis will help to determine whether a related epigenome reprogramming is responsible for the accessibility to genes in the facultative heterochromatin regions when plant species experience their development or specific environmental cues.

## Supplementary Material

gkae690_Supplemental_Files

## Data Availability

The data that support this study are available from the corresponding author upon reasonable request. Raw sequencing data generated in the course of this study have been deposited to the Gene Expression Omnibus (GEO) database under accession number GSE243911.
