# Supplementary material for: Genetic–epigenetic interplay in the determination of plant 3D genome organization
Source: Nucleic Acids Res. 2024 Aug 16;52(17):10220–34. doi: 10.1093/nar/gkae690 (PMC11417408; doi:10.1093/nar/gkae690)
Supplement: gkae690_Supplemental_Files [file gkae690_supplemental_files.zip › 5_Supplementary_figure_with_legend_high_quality.pdf]

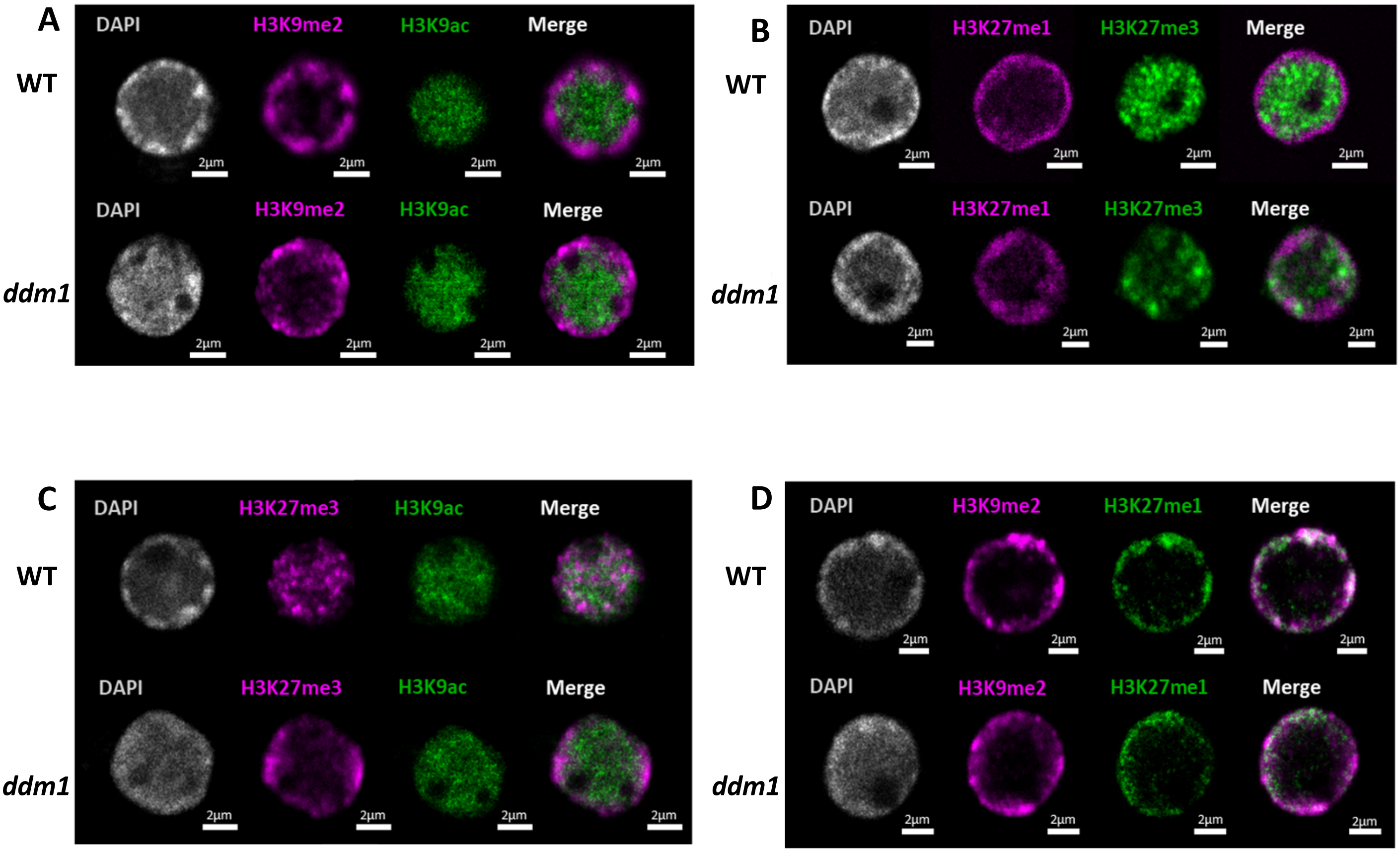

Supplemental Figure S1: **Effect of the mutation of DDM1 on chromatin organization.** (A) Immunofluorescence detection of H3K9me2 (pink), H3K9ac (green) and DAPI staining (grey) in an isolated tomato nucleus of wild type and *ddm1.* (B) Immunofluorescence detection of H3K27me1 (pink), H3K27me3 (green) and DAPI staining (grey) in an isolated tomato nucleus of wild type and *ddm1*. (C) Immunofluorescence detection of H3K27me3 (pink), H3K9ac (green) and DAPI staining (grey) in an isolated tomato nucleus of wild type and *ddm1*. (D) Immunofluorescence detection of H3K9me2 (pink), H3K27me1 (green) and DAPI staining (grey) in an isolated tomato nucleus of wild type and *ddm1*.

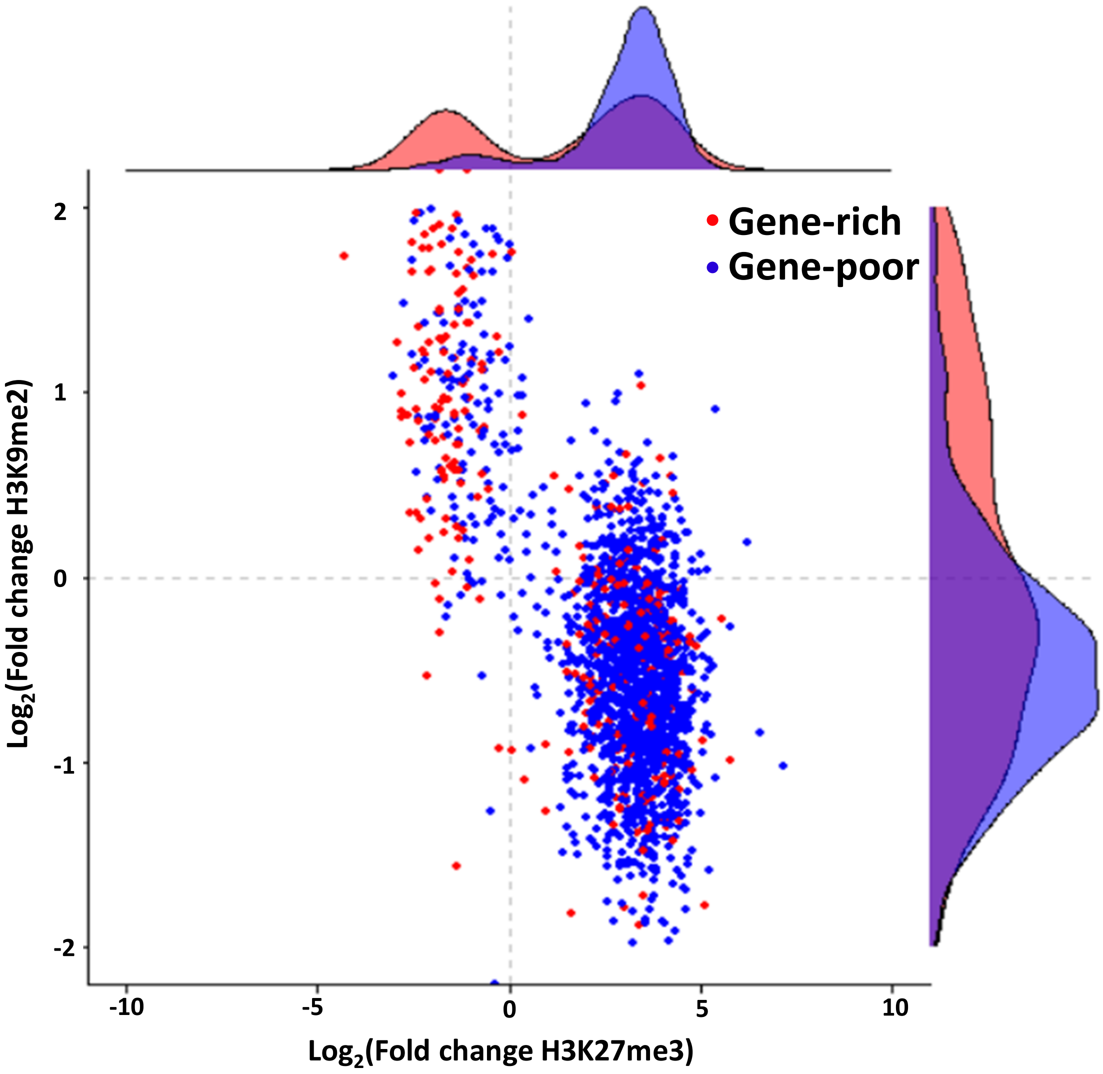

Supplemental Figure S2: Scatter plot of euchromatic TEs (red) and heterochromatic TEs (blue) with trend line according to the foldchange of H3K27me3 on x-axis and H3K9me2 on y-axis.

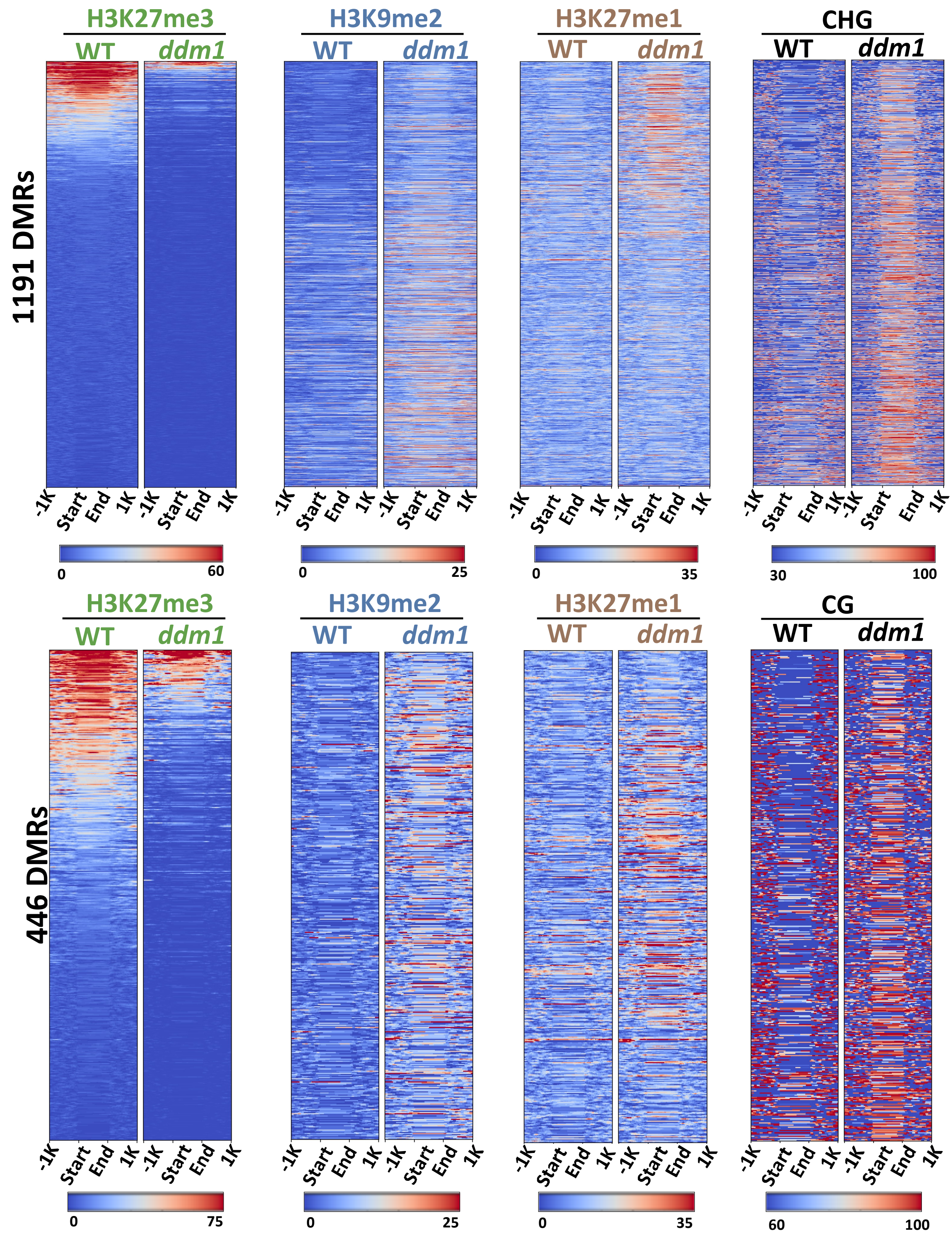

Supplemental Figure S3: Heatmaps of ChIP-seq (H3K27me3 (green), H3K9me2 (blue), H3K27me1 (brown)) and DNA methylation (CHG (black) on top and CG (black) at the bottom) from differentially methylated regions (DMRs) selected in Corem et al. (Corem et al, 2018).

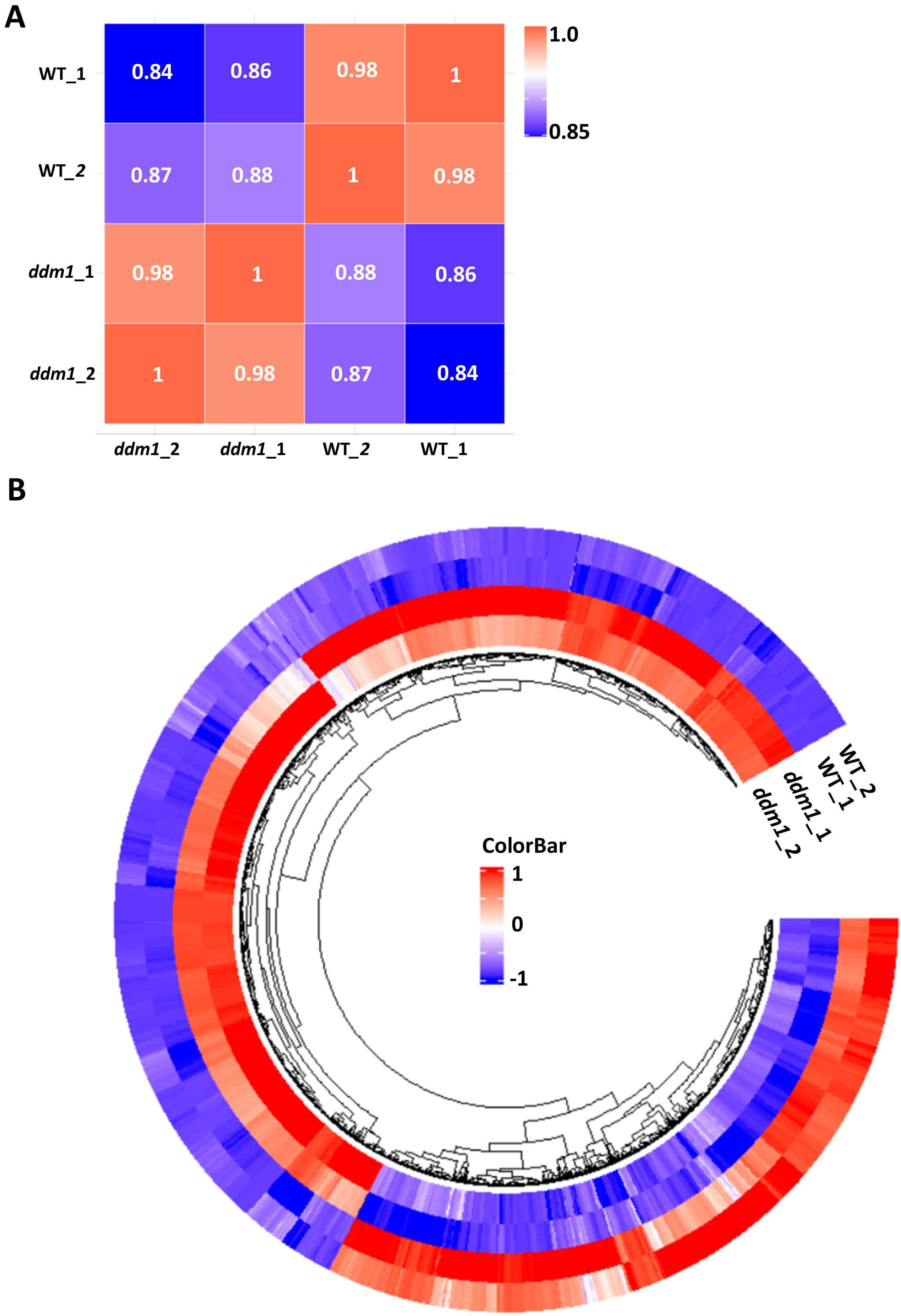

Supplemental Figure S4: **Correlation of the RNAseq data between replicates from the wild type and *ddm1*.** (A) Correlation heatmap between replicates. Color scale shows the Pearson's correlation coefficient. (B) Circos heatmap plot showing differentially expressed genes in replicates from the wild type compared to *ddm1*. Color scale shows the level of expression compared to the wild type.

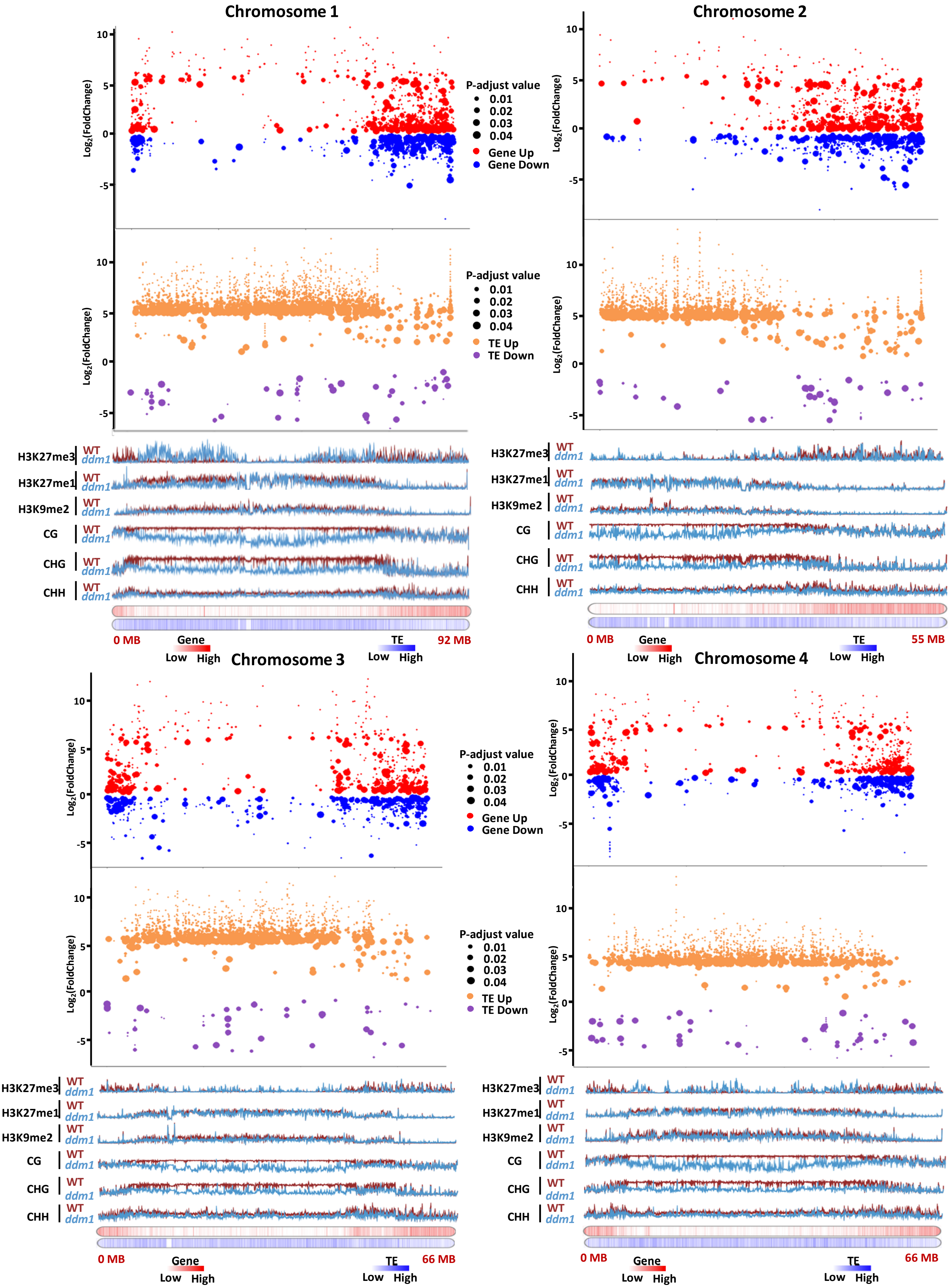

Supplemental Figure S5: Scatter plot of differentially expressed gene and TE in all the chromosome. Up-regulated gene/TE (red, orange) and down-regulated gene/TE (blue, purple) with p-value associated. ChIP-seq data (H3K27me3, H3K27me1 and H3K9me2), DNA methylation data (CG, CHG and CHH), gene and TE density are linked with the plot at the bottom of the figure, with wild type (brown) and *ddm1* (light blue).

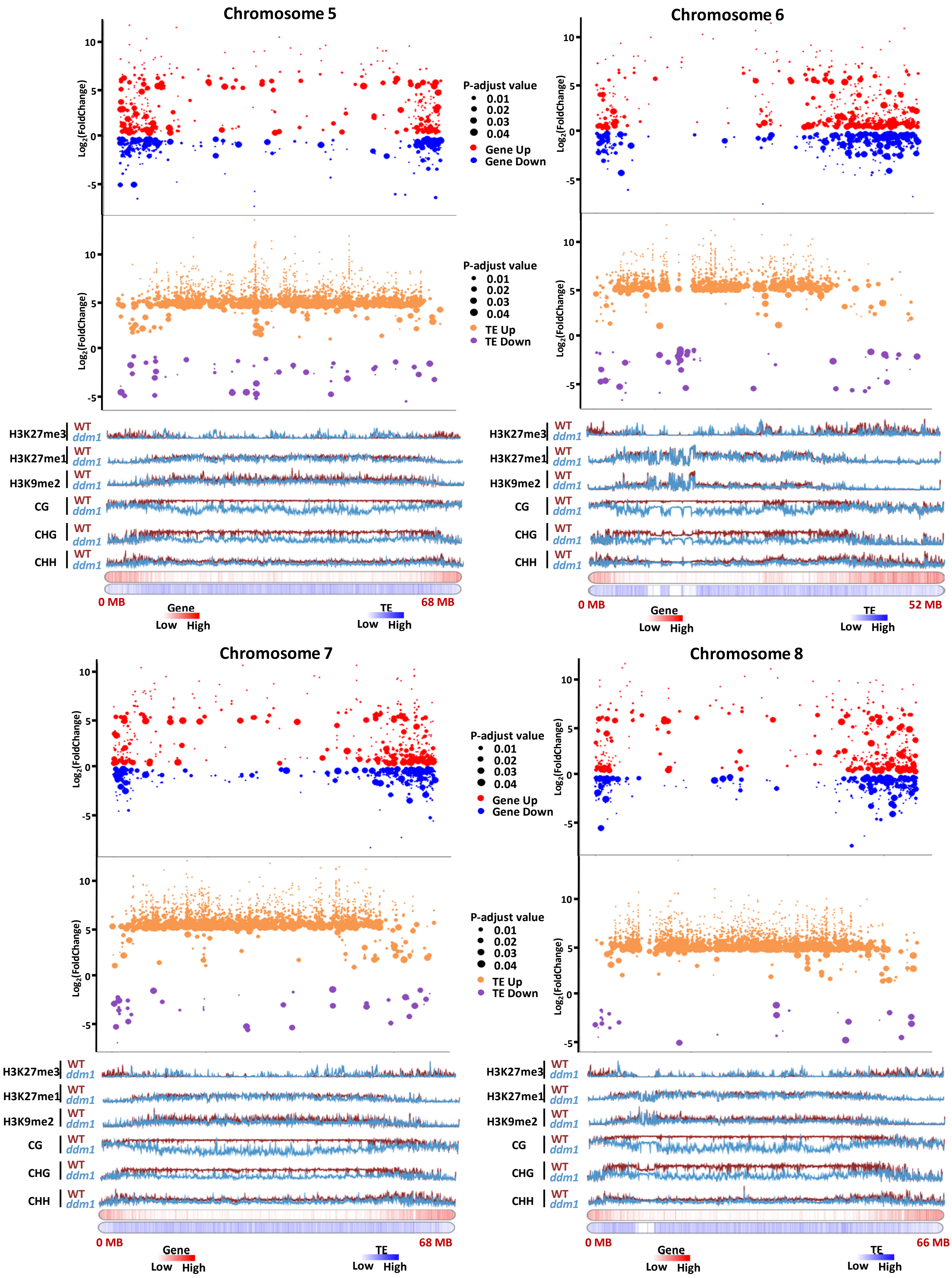

Supplemental Figure S5: Scatter plot of differentially expressed gene and TE in all the chromosome. Up-regulated gene/TE (red, orange) and down-regulated gene/TE (blue, purple) with p-value associated. ChIP-seq data (H3K27me3, H3K27me1 and H3K9me2), DNA methylation data (CG, CHG and CHH), gene and TE density are linked with the plot at the bottom of the figure, with wild type (brown) and *ddm1* (light blue).

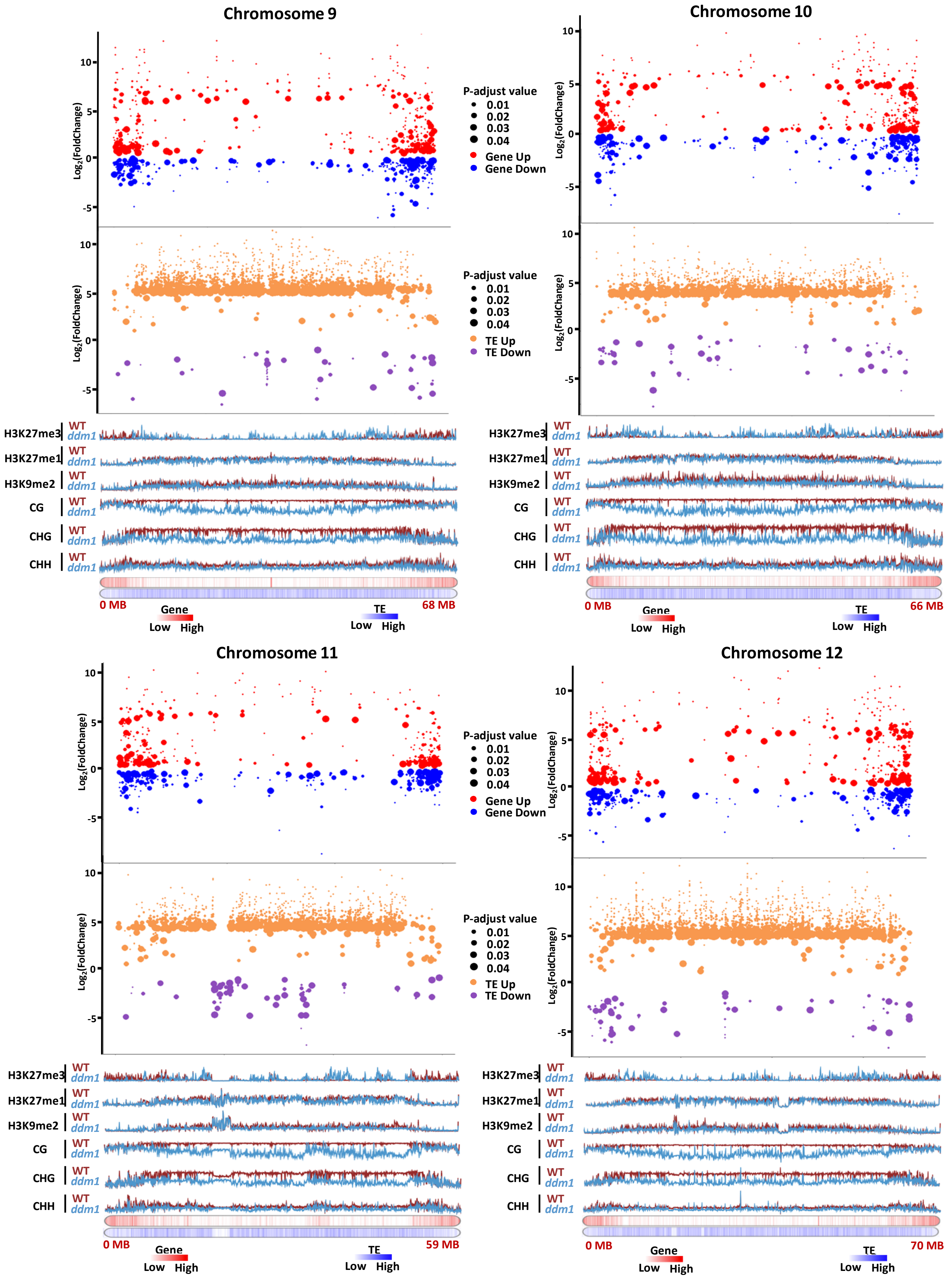

Supplemental Figure S5: Scatter plot of differentially expressed gene and TE in all the chromosome. Up-regulated gene/TE (red, orange) and down-regulated gene/TE (blue, purple) with p-value associated. ChIP-seq data (H3K27me3, H3K27me1 and H3K9me2), DNA methylation data (CG, CHG and CHH), gene and TE density are linked with the plot at the bottom of the figure, with wild type (brown) and *ddm1* (light blue).

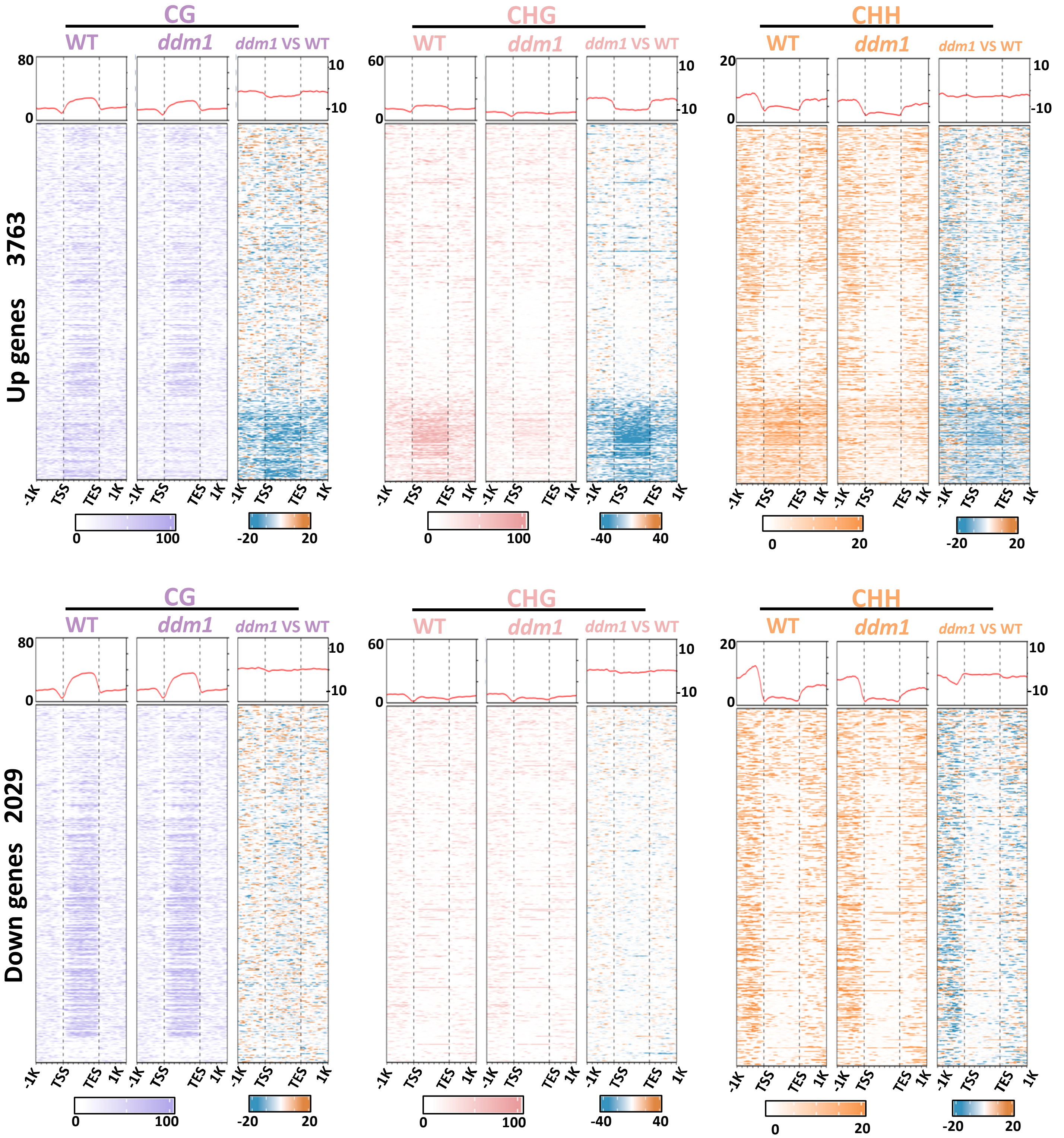

Supplemental Figure S6: Heatmaps presenting DNA methylation level depending of the cytosine context (purple for CG, pink for CHG and orange for CHH) for up-regulated genes (top) and down-regulated genes (bottom).

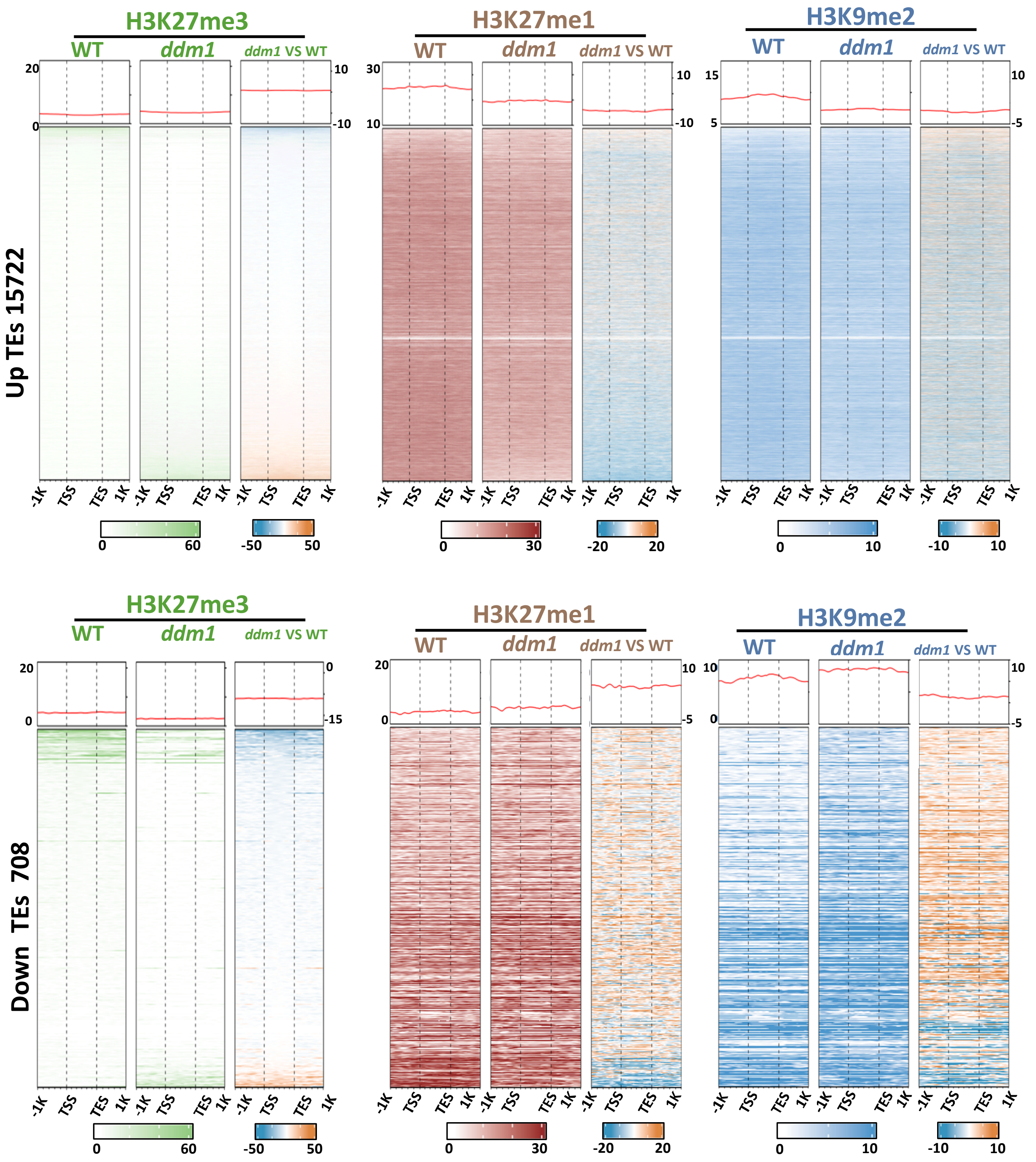

Supplemental Figure S7: **H3K27me3 focused clustered ChIP-seq heatmaps of up-regulated TEs (top) and down-regulated TEs (bottom) in the wild type and with H3K27me3 (green), H3K27me1 (brown), H3K9me2 (blue).**

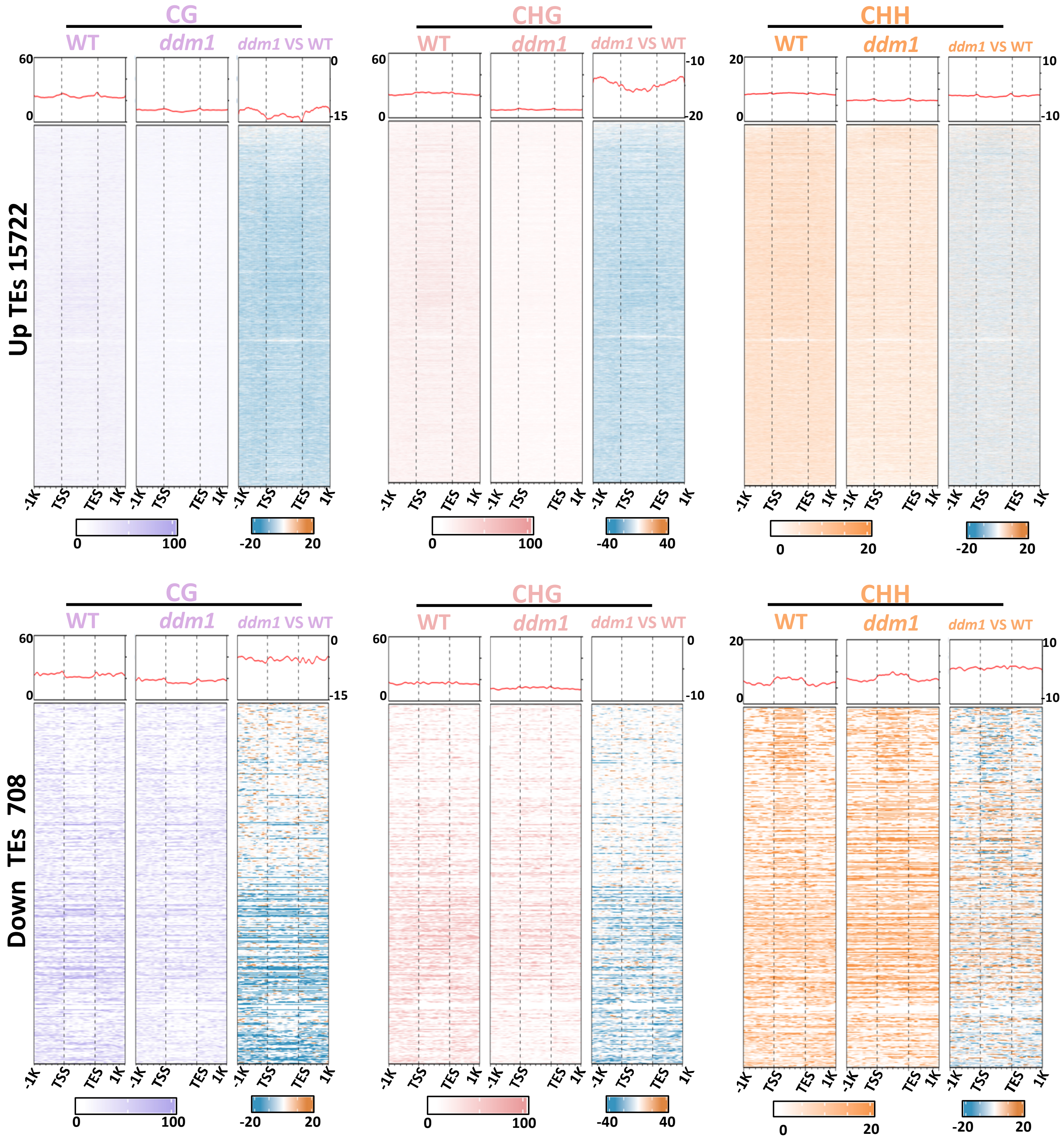

Supplemental Figure S8: Heatmaps presenting DNA methylation level depending of the cytosine context (purple for CG, pink for CHG and orange for CHH) for up-regulated genes (top) and down-regulated genes (bottom).

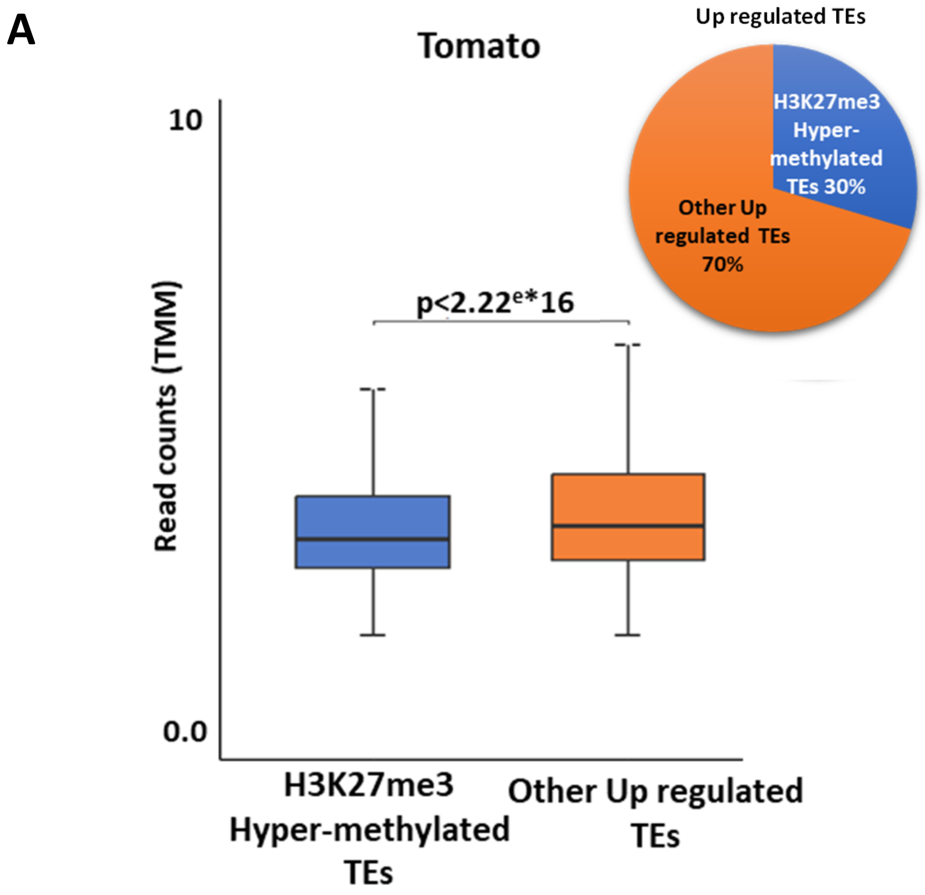

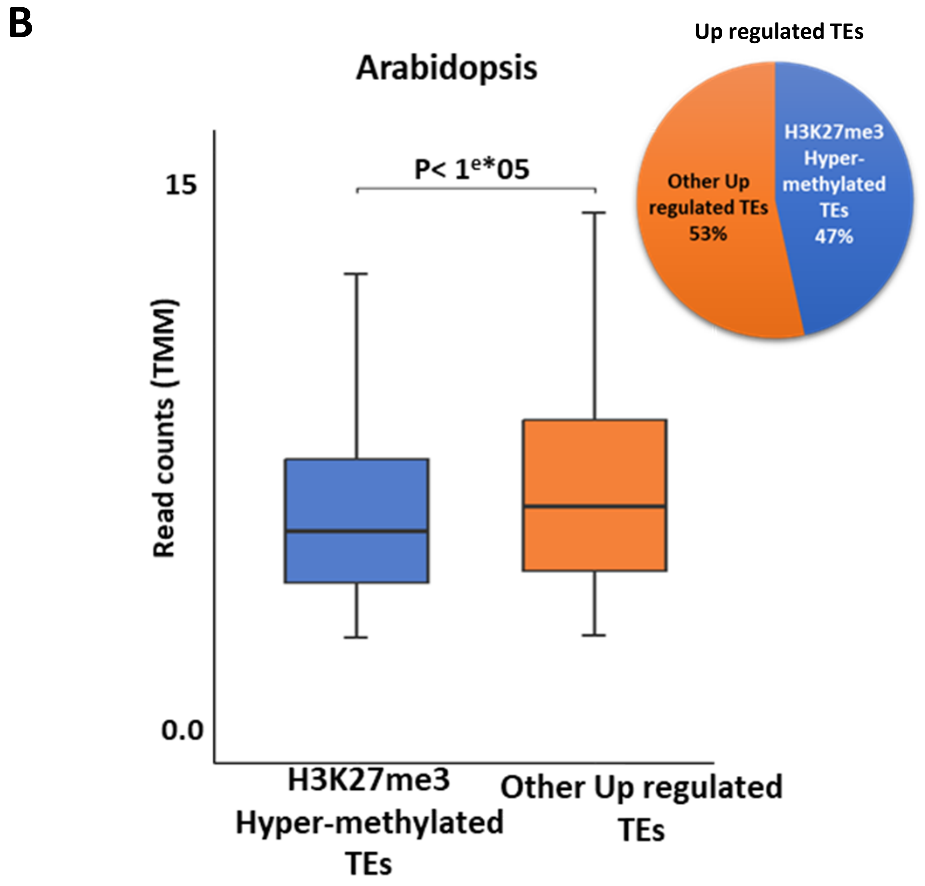

Supplemental Figure S9: **TEs marked by H3K27me3 has significant lower expression levels compared to TEs without H3K27me3 in all up-regulated TEs.** Box plot indicate Trimmed Mean of M values (TMM) normalized RNA-seq data read counts in *ddm1* mutant in tomato (A) and Arabidopsis (B). P-values calculated using Wilcoxon test. Pie charts represent the percentage of TEs with or without H3K27me3 among all up-regulated TEs.

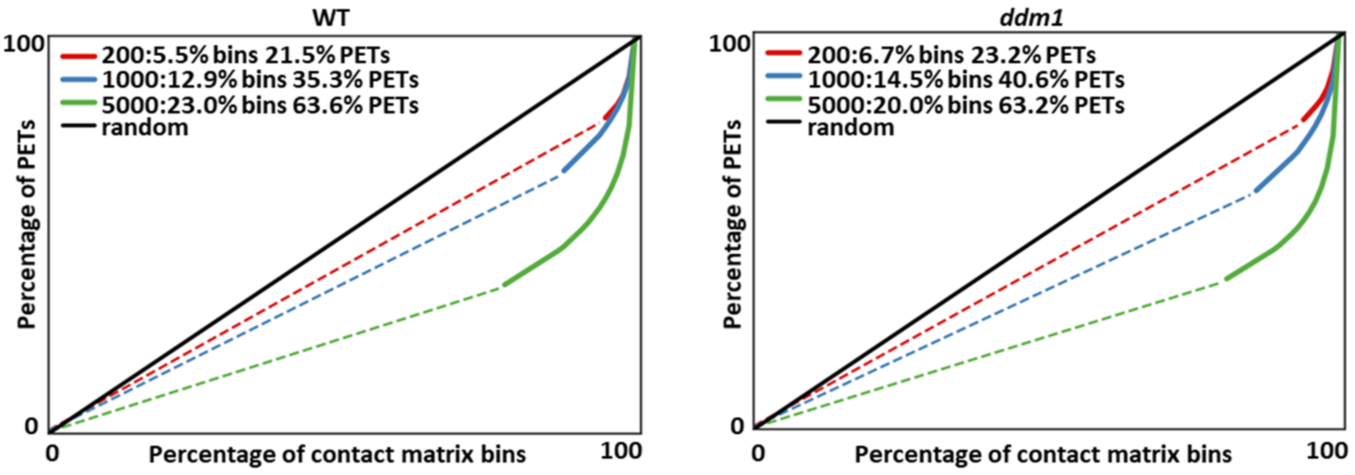

Supplemental Figure S10: **Estimation of the contact matrix resolution.** Line chart representing the percentage of paired-end tags (PETs) on the percentage of contact matrix bins for WT (left) and *ddm1* (right). The dashed line shows the bins from the contact matrix with only singleton PET, and the solid lines show the bins with multiple PETs. The random line indicates all PETs are evenly distributed in the contact matrix bins. Generated with cLoops2 (Cao et al., 2022).

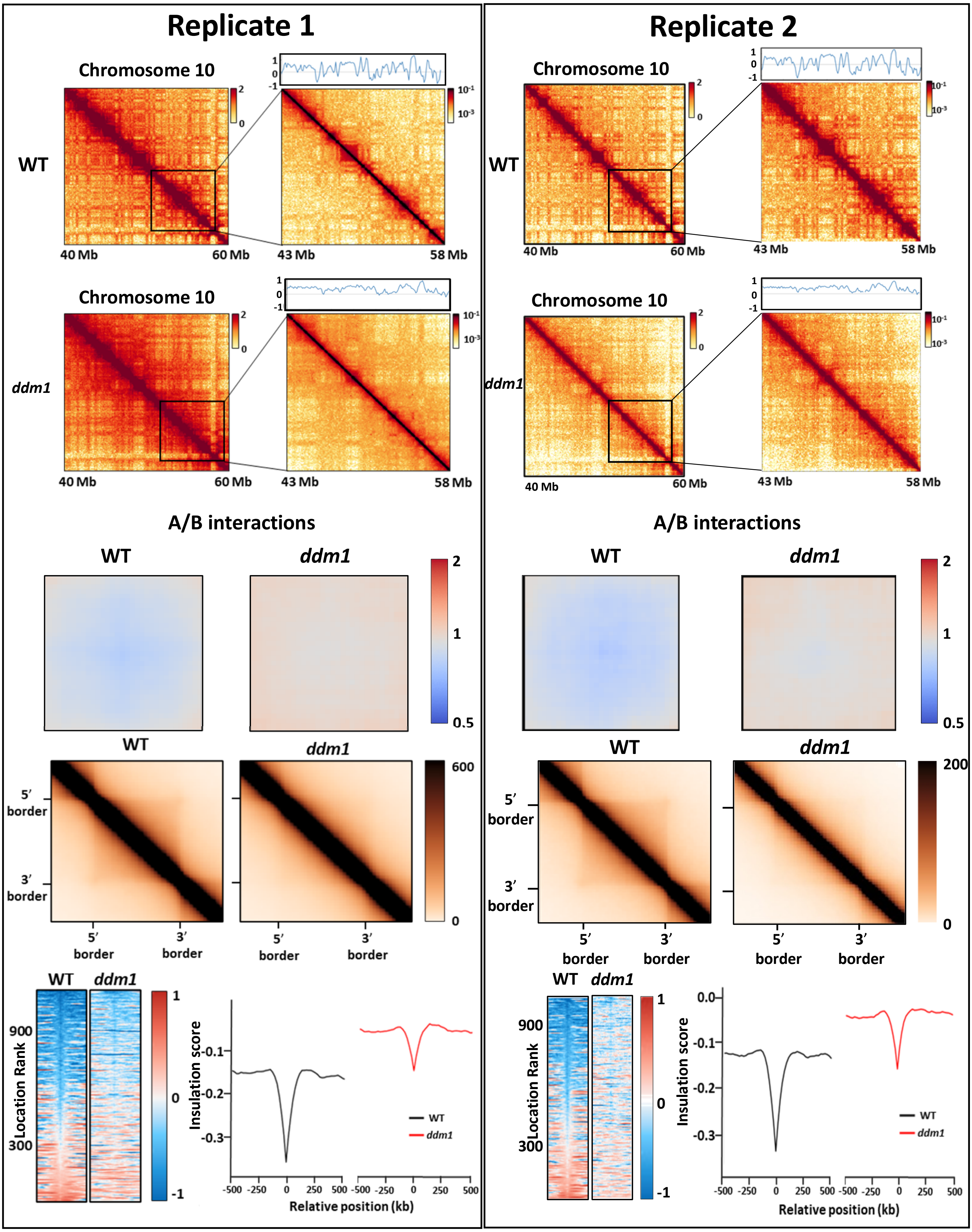

Supplemental Figure S11: Replicates of the Hi-C interaction matrix, Pentad analysis plots, aggregate TAD plots, the insulation heatmaps and the graphs of the insulation score versus the relative position in kb of the chromosome 10.

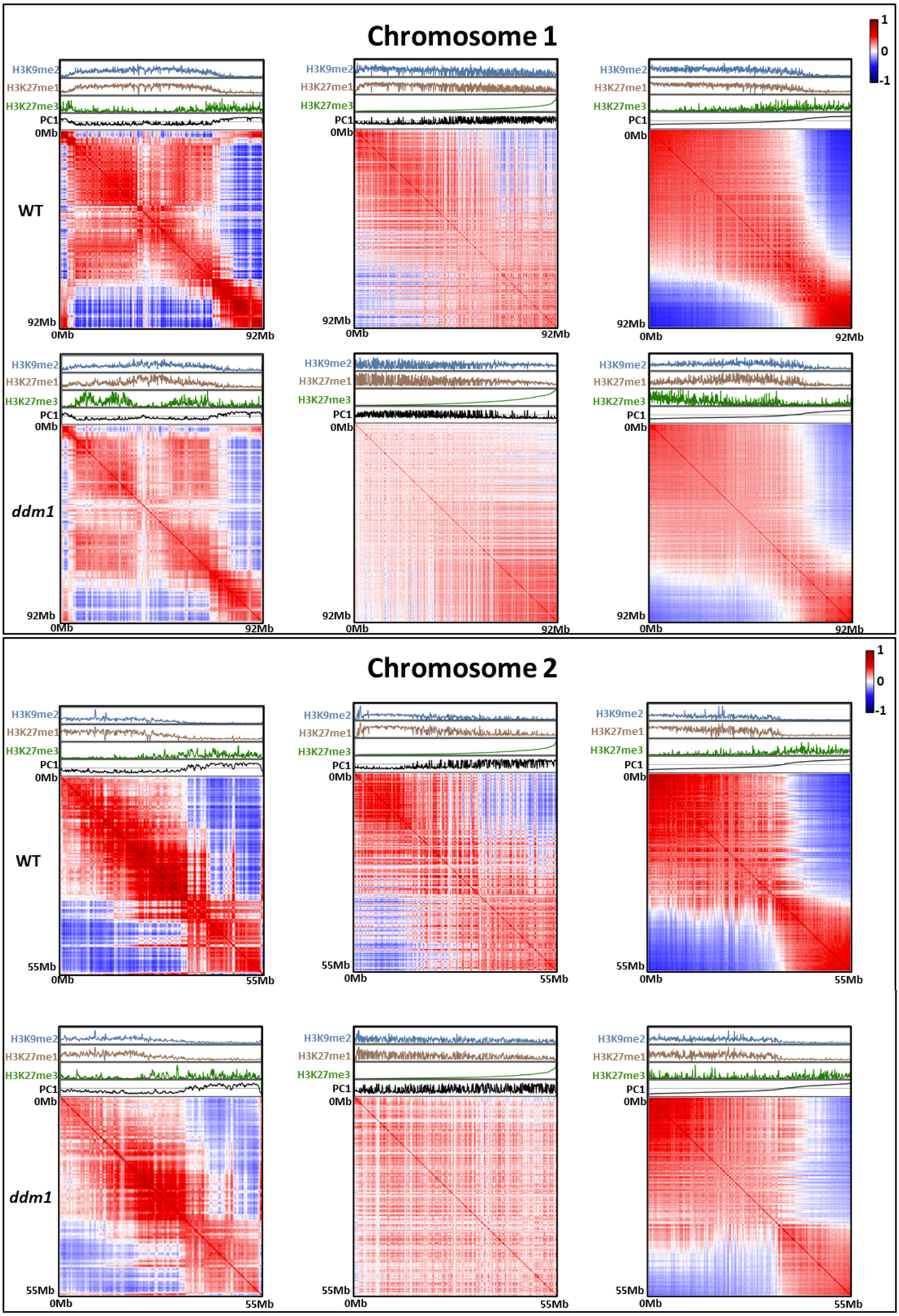

Supplemental Figure S12: Pearson correlations of distance-normalized Hi-C interaction frequency maps (left) with sorted maps for H3K27me3 (middle) and PC1 (right). For chromosomes 1 and 2.

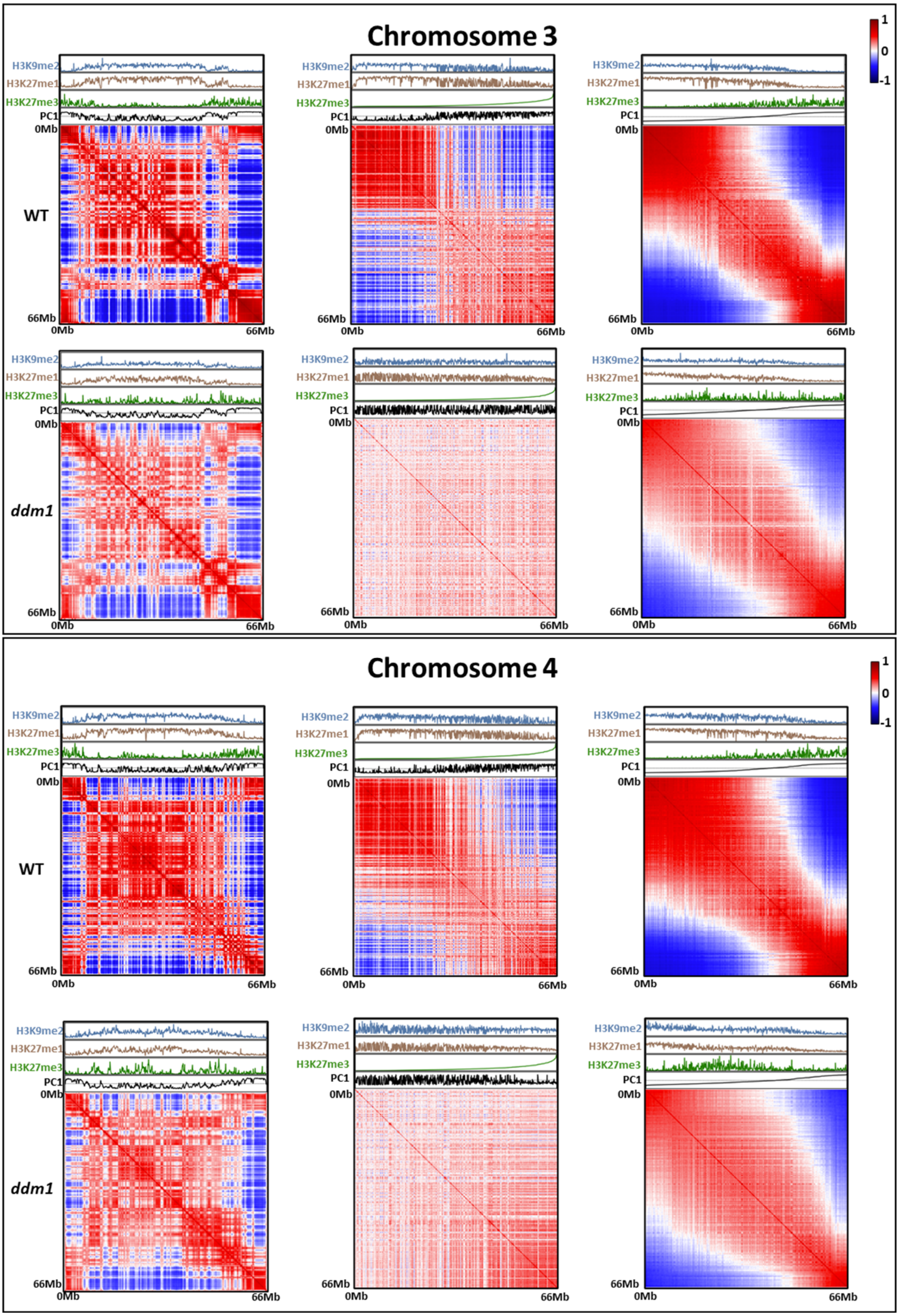

Supplemental Figure S12: Pearson correlations of distance-normalized Hi-C interaction frequency maps (left) with sorted maps for H3K27me3 (middle) and PC1 (right). For chromosomes 3 and 4.

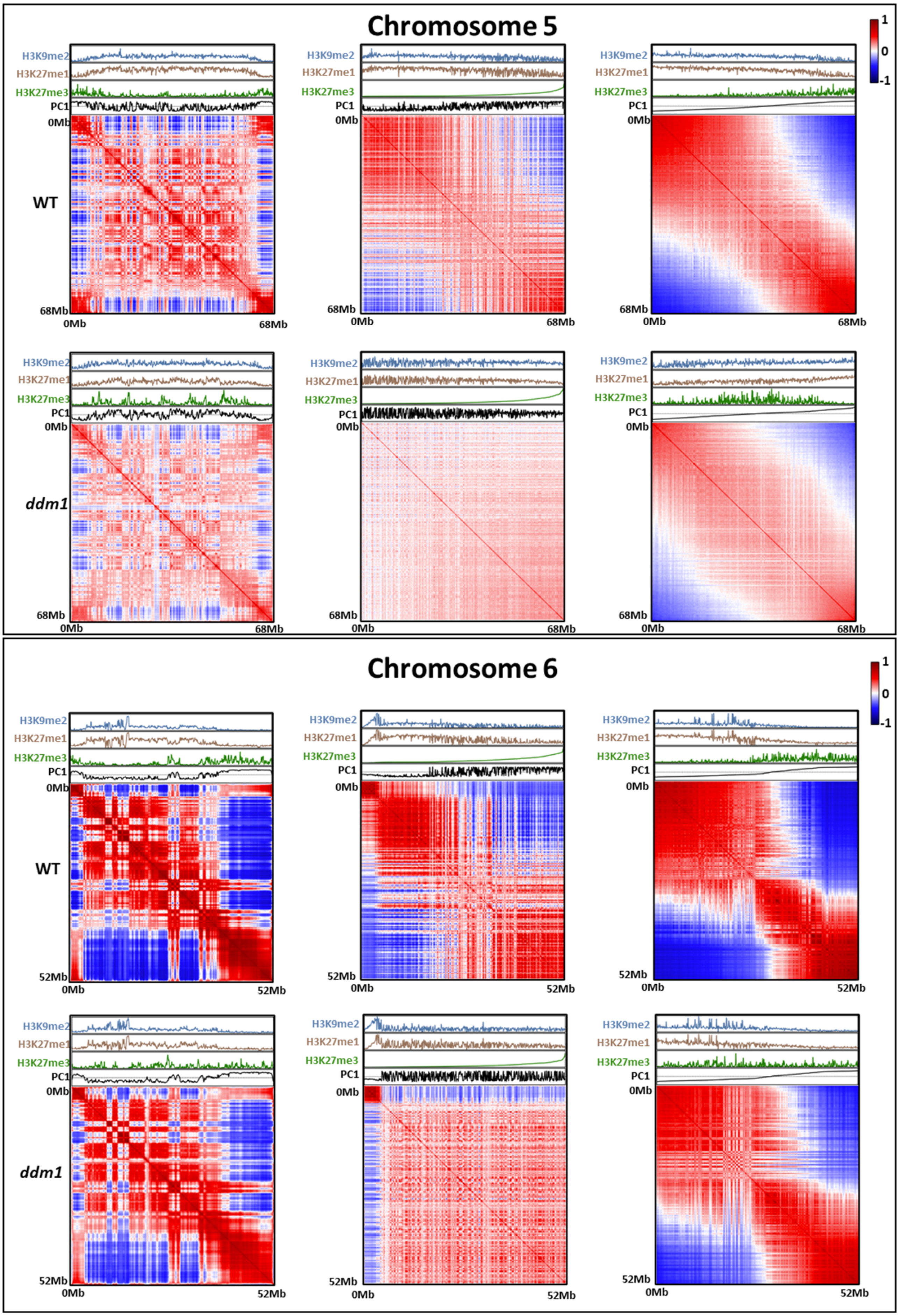

Supplemental Figure S12: **Pearson correlations of distance-normalized Hi-C interaction frequency maps (left) with sorted maps for H3K27me3 (middle) and PC1 (right).** For chromosomes 5 and 6.

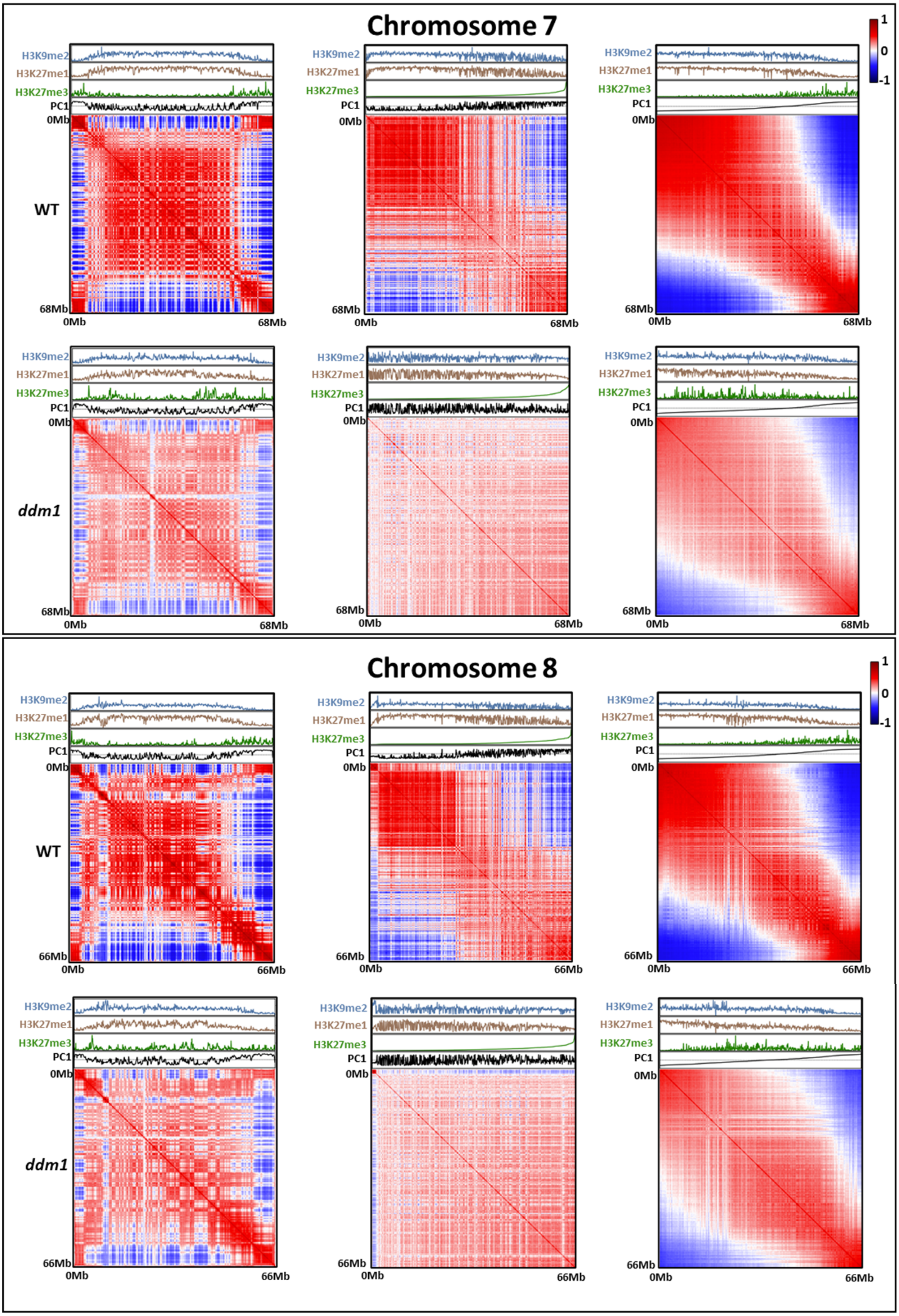

Supplemental Figure S12: Pearson correlations of distance-normalized Hi-C interaction frequency maps (left) with sorted maps for H3K27me3 (middle) and PC1 (right). For chromosomes 7 and 8.

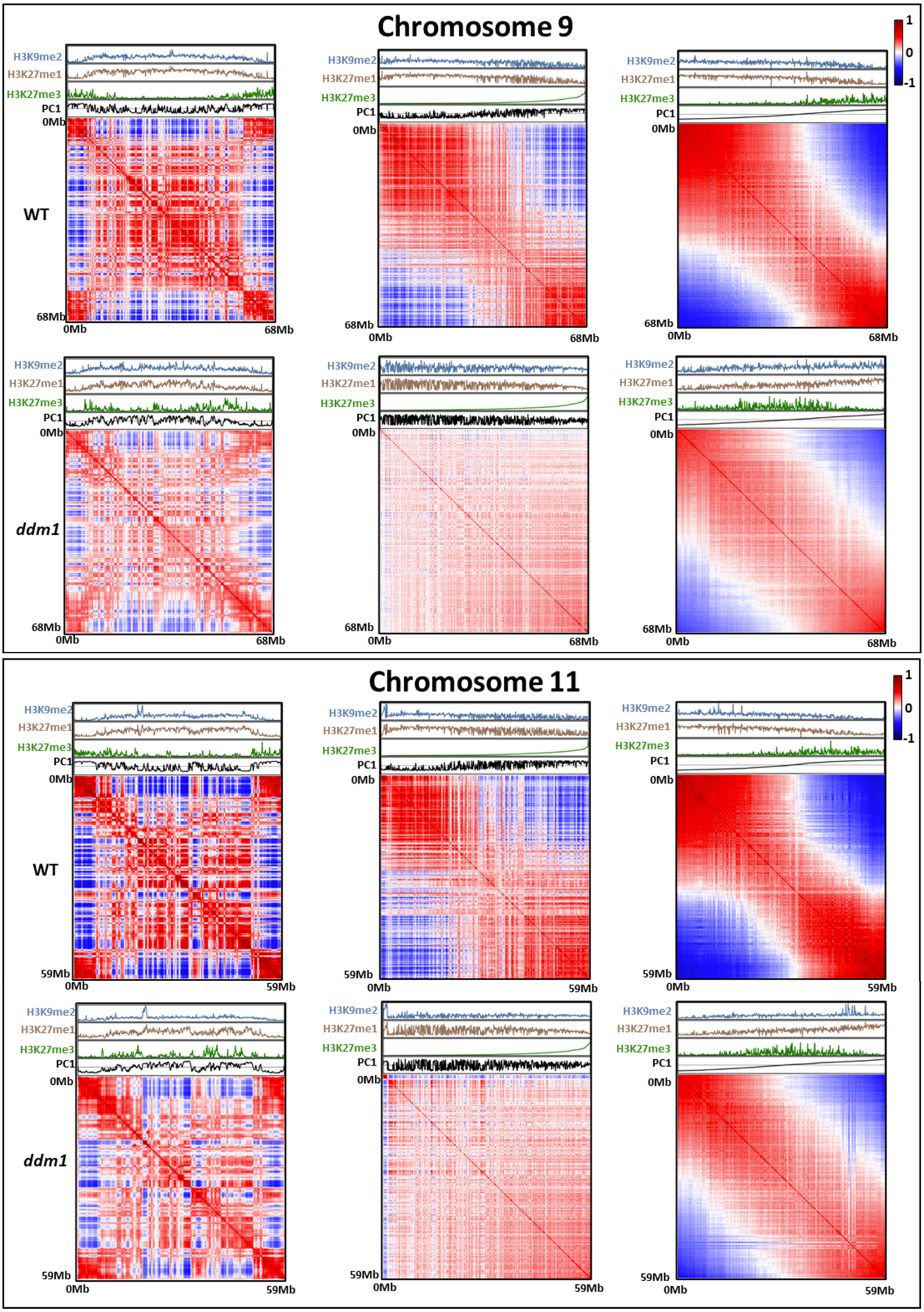

Supplemental Figure S12: **Pearson correlations of distance-normalized Hi-C interaction frequency maps (left) with sorted maps for H3K27me3 (middle) and PC1 (right).** For chromosomes 9 and 10.

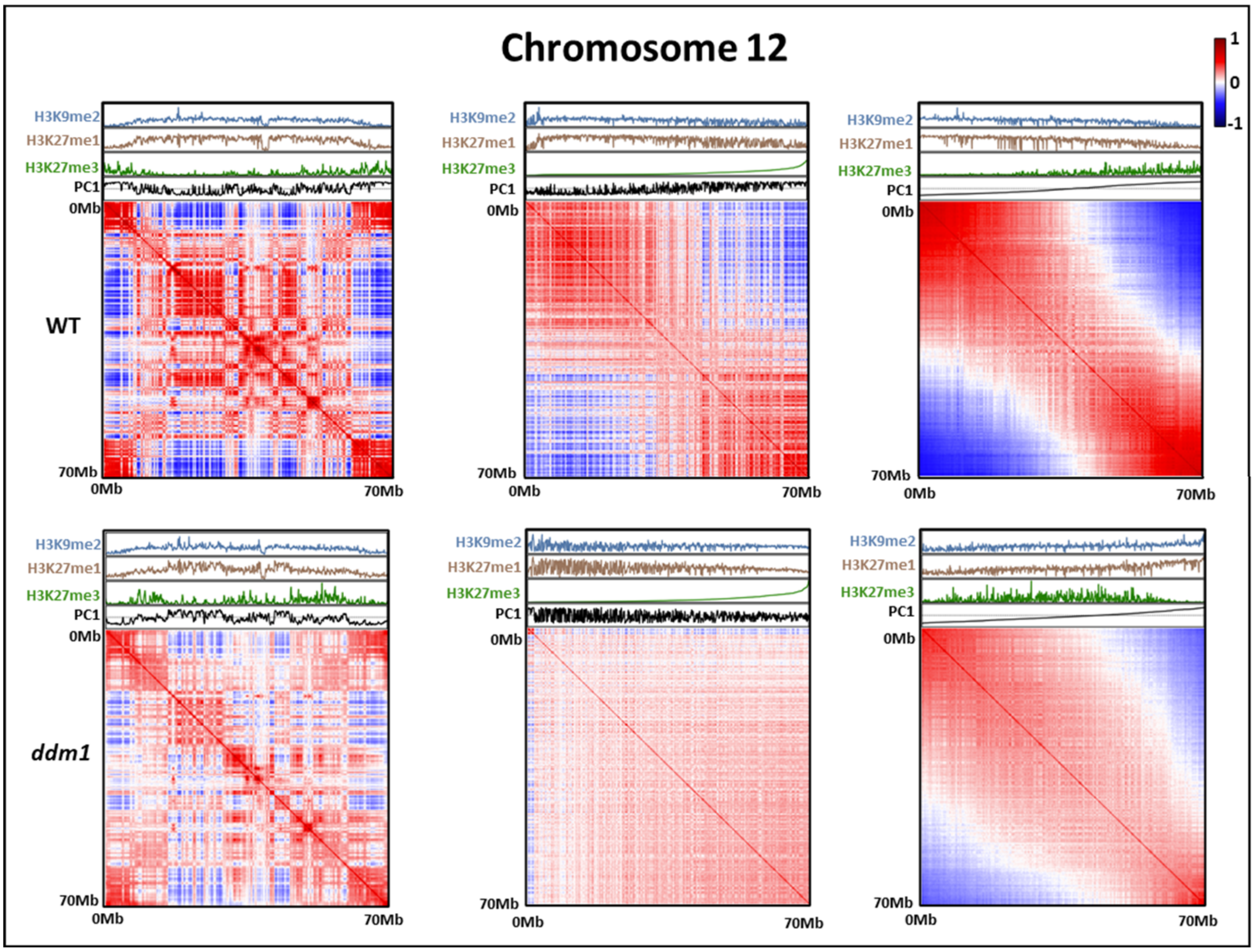

Supplemental Figure S12: **Pearson correlations of distance-normalized Hi-C interaction frequency maps (left) with sorted maps for H3K27me3 (middle) and PC1 (right).** For chromosome 12.

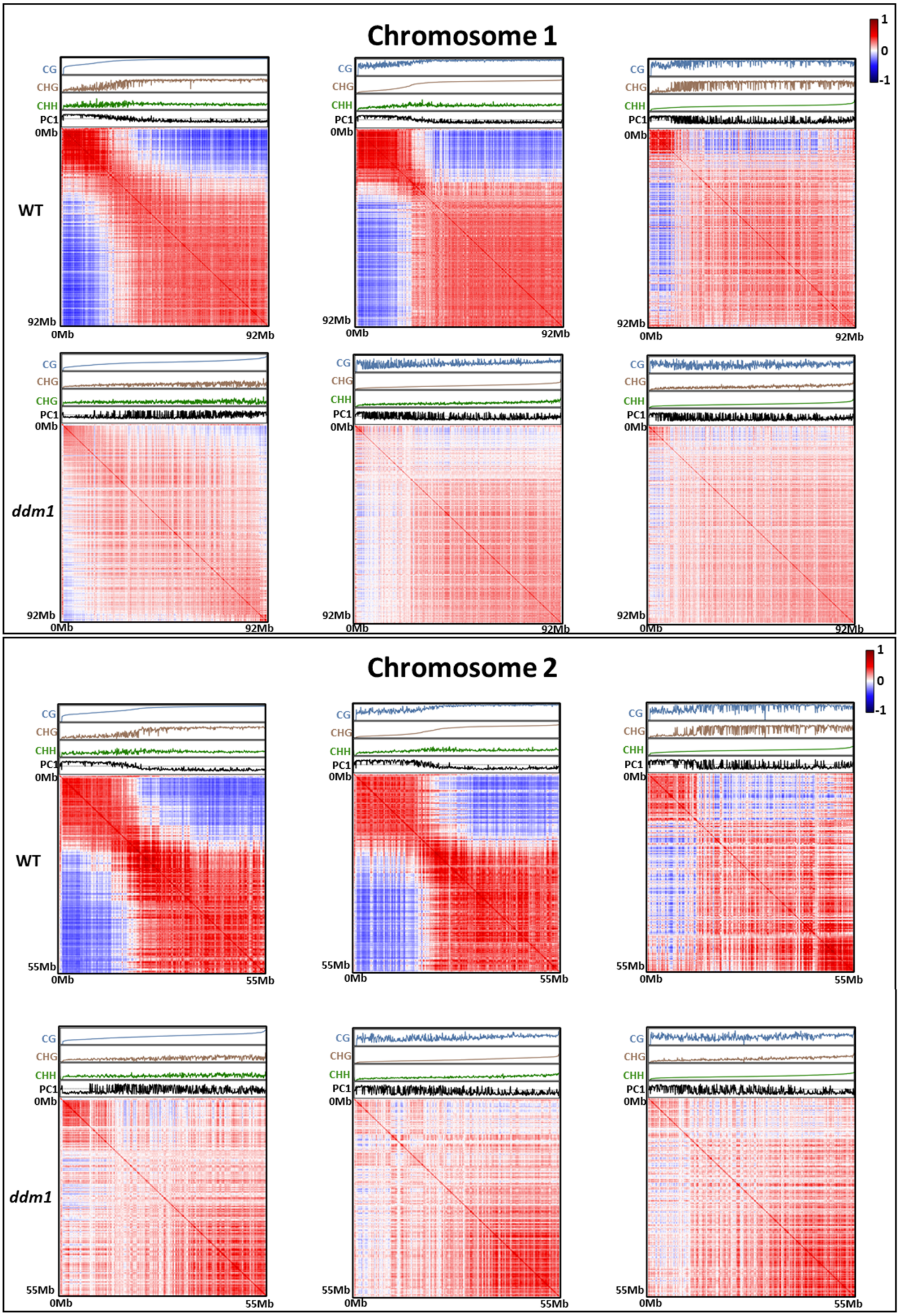

Supplemental Figure S13: **Pearson correlations of distance-normalized Hi-C interaction frequency sorted maps for CG (left), CHG (middle) and CHH (right).** For chromosomes 1 and 2.

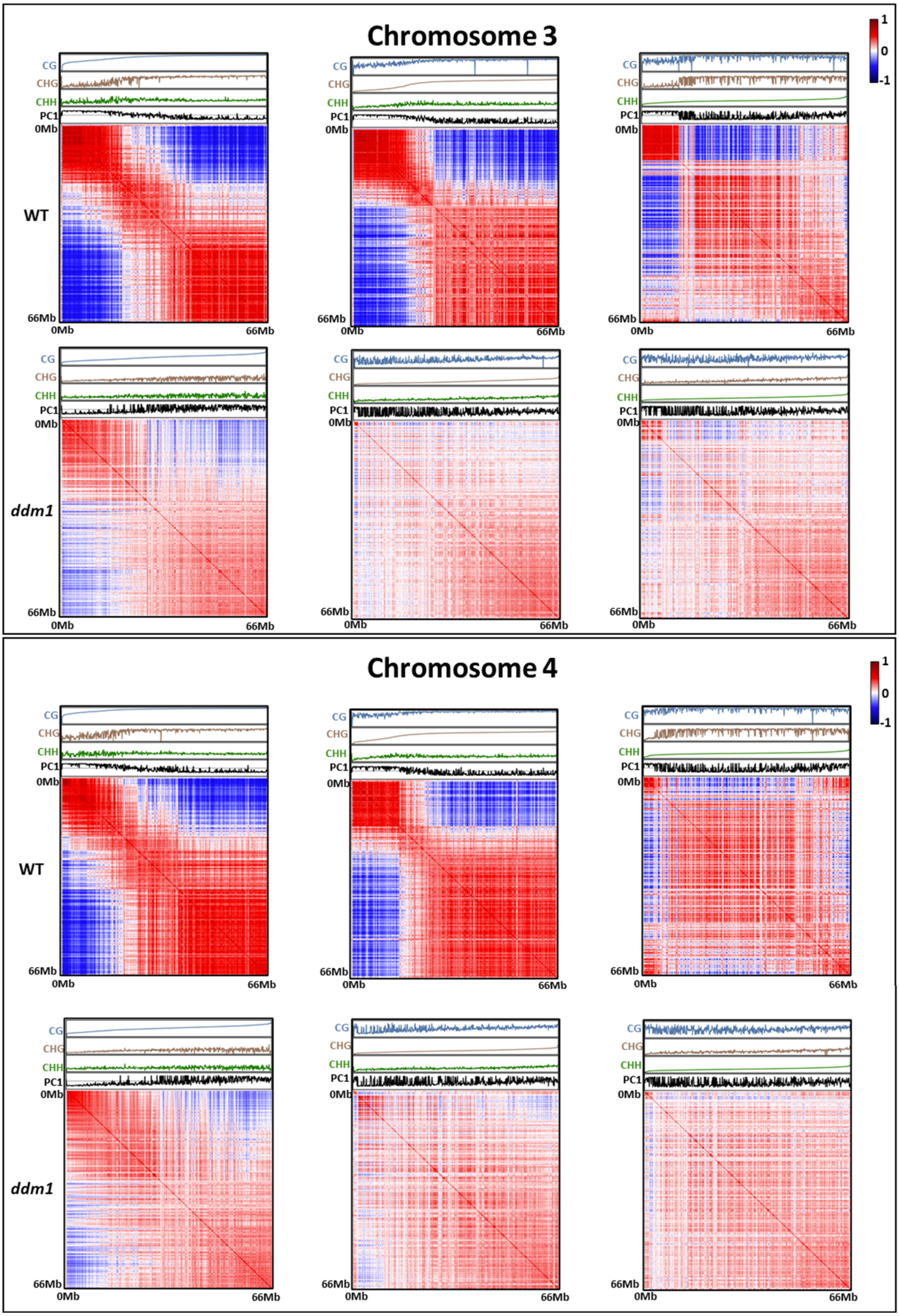

Supplemental Figure S13: **Pearson correlations of distance-normalized Hi-C interaction frequency sorted maps for CG (left), CHG (middle) and CHH (right).** For chromosomes 3 and 4.

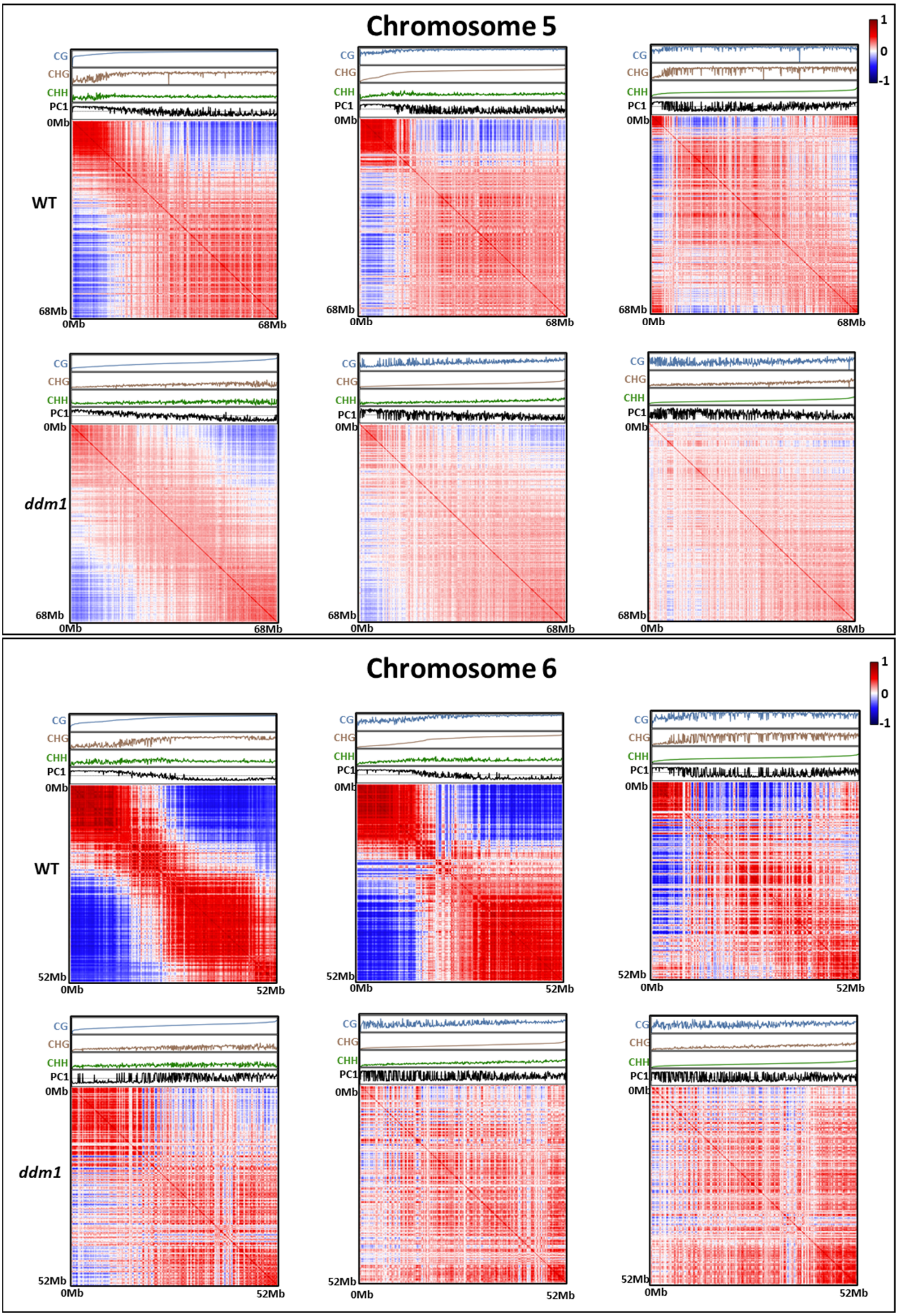

Supplemental Figure S13: **Pearson correlations of distance-normalized Hi-C interaction frequency sorted maps for CG (left), CHG (middle) and CHH (right).** For chromosomes 5 and 6.

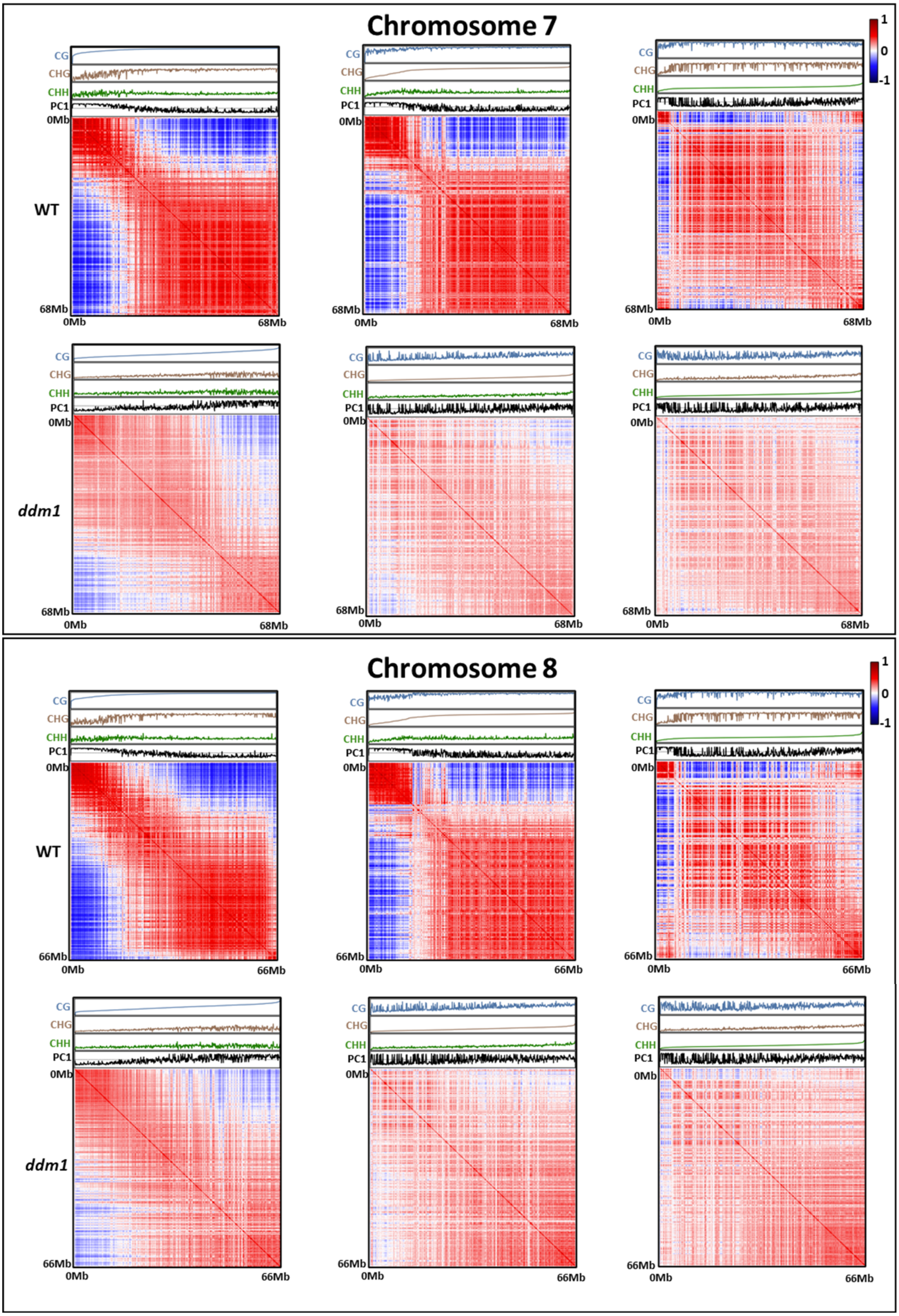

Supplemental Figure S13: **Pearson correlations of distance-normalized Hi-C interaction frequency sorted maps for CG (left), CHG (middle) and CHH (right).** For chromosomes 7 and 8.

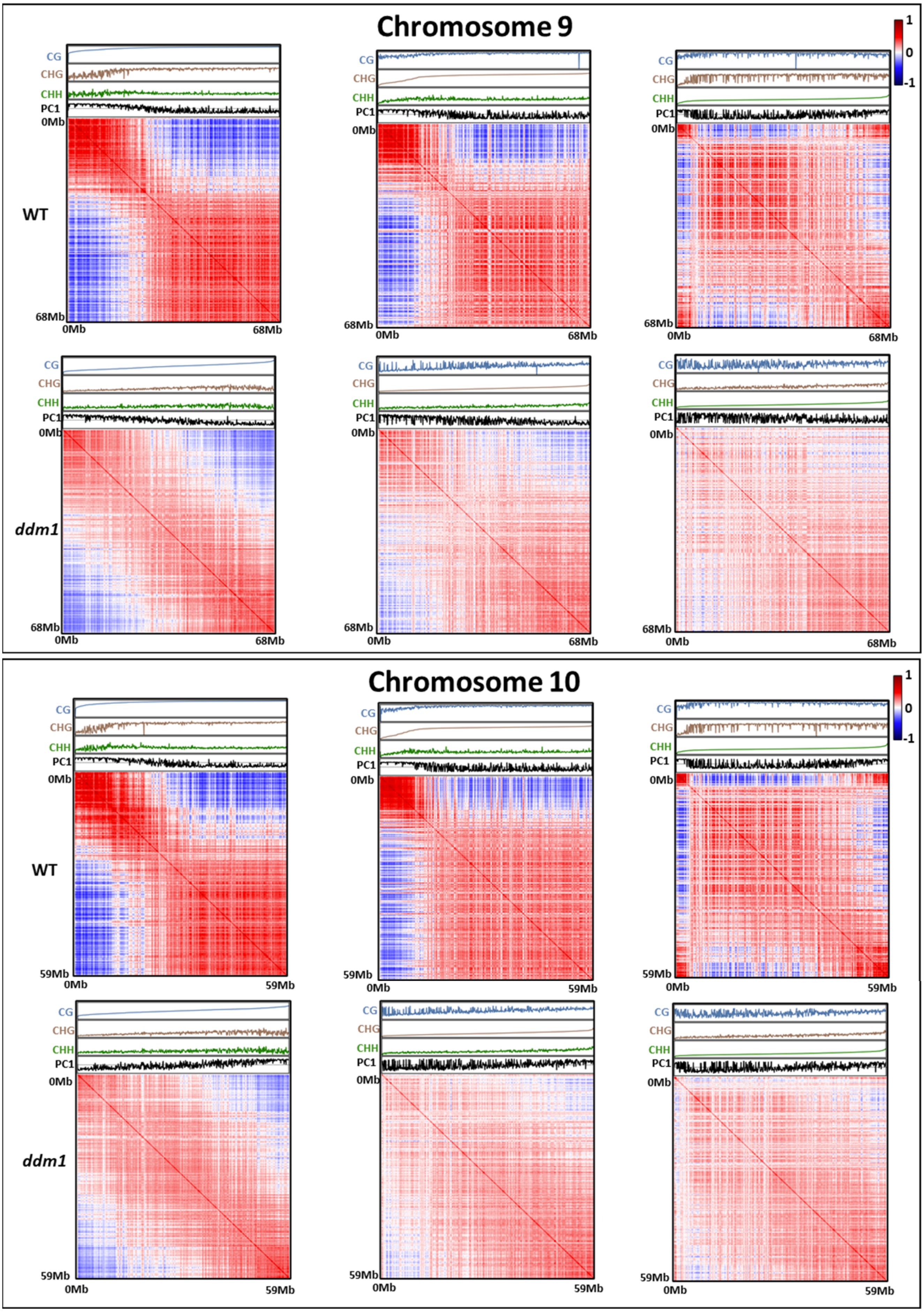

Supplemental Figure S13: **Pearson correlations of distance-normalized Hi-C interaction frequency sorted maps for CG (left), CHG (middle) and CHH (right).** For chromosomes 9 and 10.

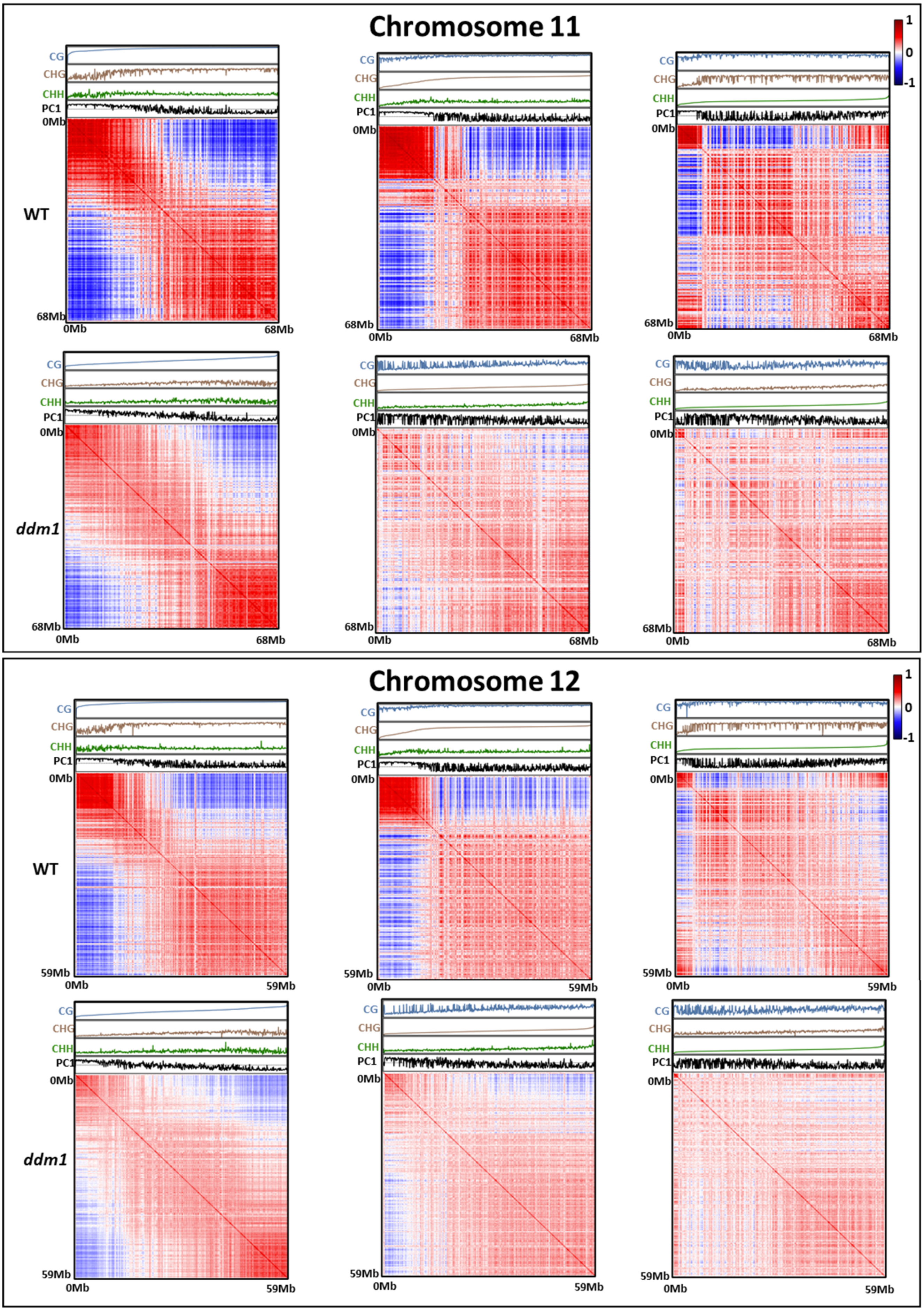

Supplemental Figure S13: **Pearson correlations of distance-normalized Hi-C interaction frequency sorted maps for CG (left), CHG (middle) and CHH (right).** For chromosomes 11 and 12.

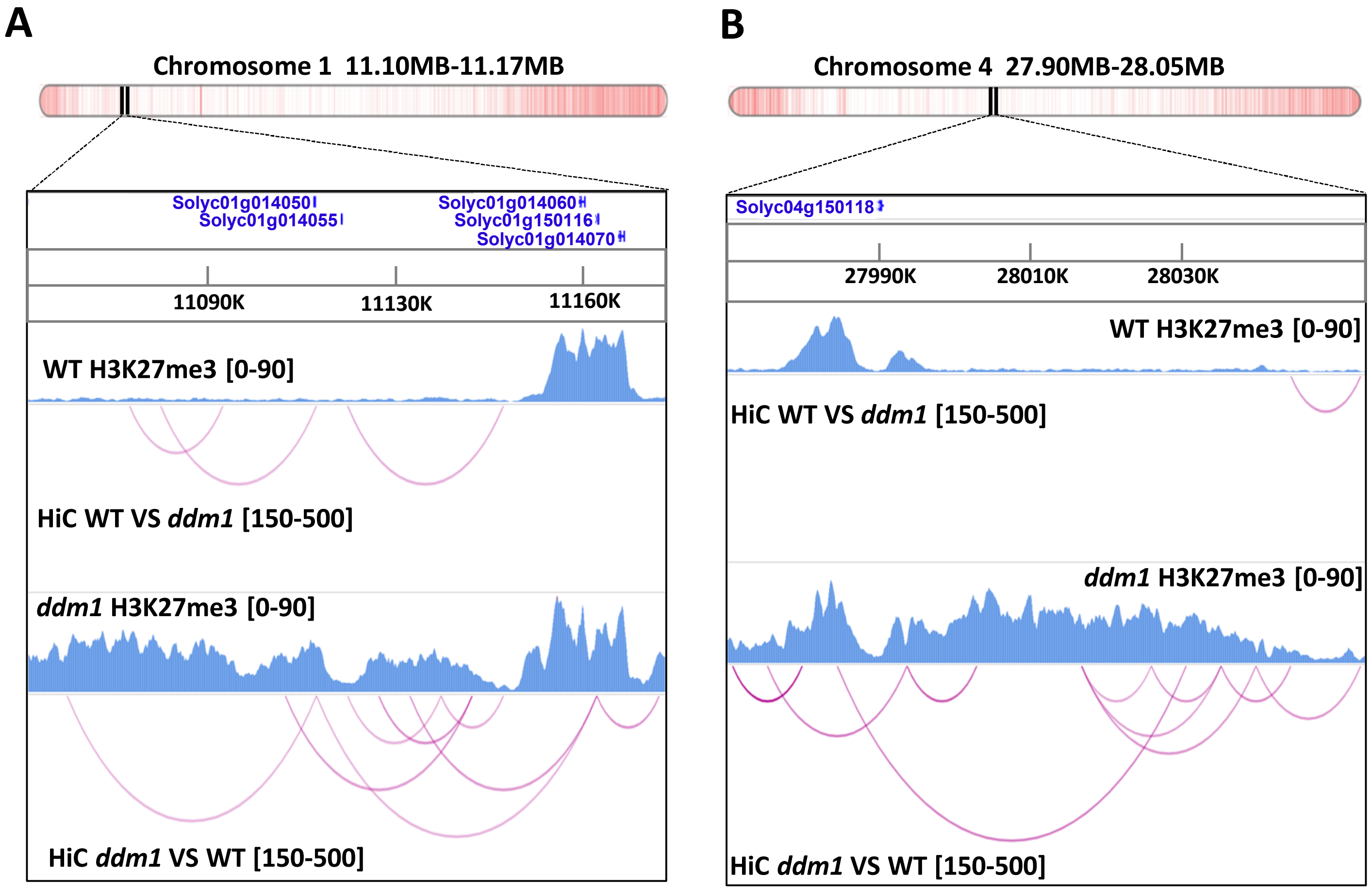

Supplemental Figure S14: **Interactions plots for 2 genes representing wild type at the top and *ddm1* at the bottom.** Genes and H3K27me3 ChIP-seq data are aligned with the interactions arcs. The corresponding chromosome is indicated at the top of the plot with the relative gene density, with a the gene-poor regions in white and the gene-rich regions in red. Interaction arc intervals were chosen according to the most significant Zscores corresponding to WT-specific interaction on the top, and *ddm1*-specific interactions at the bottom.

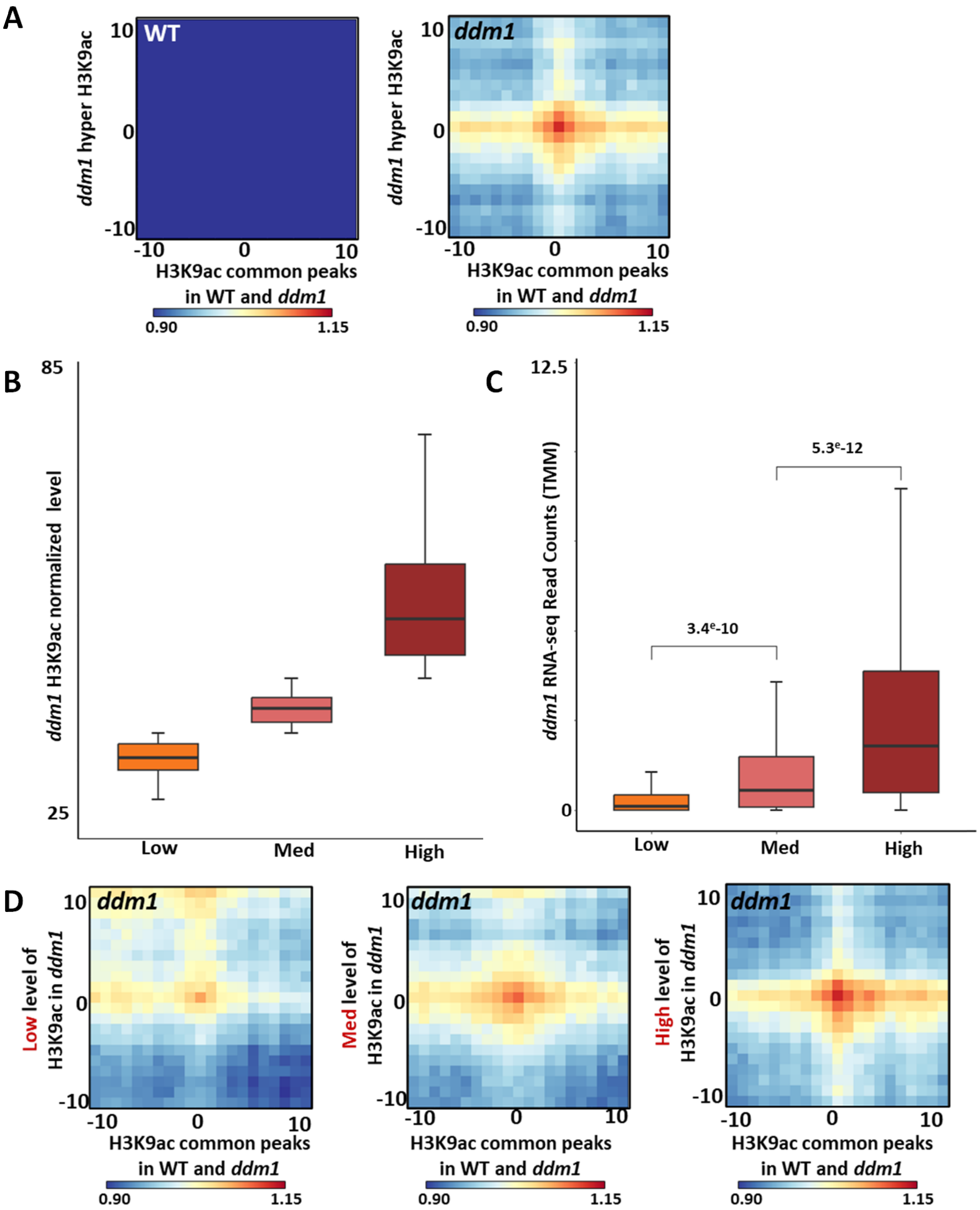

Supplemental Figure S15: **H3K9ac induces new interactions leading to the increase of gene expression in *ddm1*.** (A) Aggregate plots quantifying the mean of aggregated WT and *ddm1* Hi-C contacts matrices between regions with an increase of H3K9ac in *ddm1* and regions presenting stable peaks of H3K9ac in WT and *ddm1*. (B) Box plots indicating the levels of ectopic H3K9ac enrichment in *ddm1*, classified from low to high. (C) Box plots indicating Trimmed Mean of M values (TMM) of normalized RNA-seq data read counts for the regions that gain H3K9ac in *ddm1* mutant in 3 categories (low, medium and high level). P-values were calculated using a Wilcoxon test. (D) Aggregate plots quantifying the mean of aggregated *ddm1* Hi-C contacts matrices between regions marked with H3K9ac in *ddm1*, according to 3 levels of H3K9ac (low, medium and high) and regions with stable peaks of H3K9ac in WT and *ddm1*.
